# Grammar-constrained decoding for structured information extraction with fine-tuned generative models applied to clinical trial abstracts

**DOI:** 10.3389/frai.2024.1406857

**Published:** 2025-01-07

**Authors:** David M. Schmidt, Philipp Cimiano

**Affiliations:** Center for Cognitive Interaction Technology (CITEC), Technical Faculty, Bielefeld University, Bielefeld, Germany

**Keywords:** grammar-constrained decoding, structured information extraction, clinical trials, deep learning, generative large language models, PICO, evidence-based medicine

## Abstract

**Background:**

In the field of structured information extraction, there are typically semantic and syntactic constraints on the output of information extraction (IE) systems. These constraints, however, can typically not be guaranteed using standard (fine-tuned) encoder-decoder architectures. This has led to the development of constrained decoding approaches which allow, e.g., to specify constraints in form of context-free grammars. An open question is in how far an IE system can be effectively guided by a domain-specific grammar to ensure that the output structures follow the requirements of a certain domain data model.

**Methods:**

In this work we experimentally investigate the influence of grammar-constrained decoding as well as pointer generators on the performance of a domain-specific information extraction system. For this, we consider fine-tuned encoder-decoder models, Longformer and Flan-T5 in particular, and experimentally investigate whether the addition of grammar-constrained decoding and pointer generators improve information extraction results. Toward this goal, we consider the task of inducing structured representations from abstracts describing clinical trials, relying on the C-TrO ontology to semantically describe the clinical trials and their results. We frame the task as a slot filling problem where certain slots of templates need to be filled with token sequences occurring in the input text. We use a dataset comprising 211 annotated clinical trial abstracts about type 2 diabetes and glaucoma for training and evaluation. Our focus is on settings in which the available training data is in the order of a few hundred training examples, which we consider as a *low-resource setting*.

**Results:**

In all our experiments we could demonstrate the positive impact of grammar-constrained decoding, with an increase in *F*_1_ score of pp 0.351 (absolute score 0.413) and pp 0.425 (absolute score 0.47) for the best-performing models on type 2 diabetes and glaucoma datasets, respectively. The addition of the pointer generators had a detrimental impact on the results, decreasing *F*_1_ scores by pp 0.15 (absolute score 0.263) and pp 0.198 (absolute score 0.272) for the best-performing pointer generator models on type 2 diabetes and glaucoma datasets, respectively.

**Conclusion:**

The experimental results indicate that encoder-decoder models used for structure prediction for information extraction tasks in low-resource settings clearly benefit from grammar-constrained decoding guiding the output generation. In contrast, the evaluated pointer generator models decreased the performance drastically in some cases. Moreover, the performance of the pointer models appears to depend both on the used base model as well as the function used for aggregating the attention values. How the size of large language models affects the performance benefit of grammar-constrained decoding remains to be more structurally investigated in future work.

## 1 Introduction

The increasing success of large language models on a wide range of tasks together with their wide availability has inspired a number of approaches in structured information extraction as well as other tasks requiring a structured prediction, e.g., event extraction (Lu et al., 2021), syntactic and semantic parsing (Roy et al., 2024), symbolic expression generation like SMT formulas (Pan et al., 2023; Sun et al., 2023) or SQL query generation from text (Scholak et al., 2021; Lin et al., 2020).

With structured output, we refer to output structures that go beyond a linear sequence, that is, representing a tree, graph or some other kind of nested structure as output. Many application areas have strict requirements on the structure of the corresponding output and it is key to ensure that the output is valid w.r.t. some pre-defined data model. However, the validity w.r.t. those constraints on the output sequence typically cannot be guaranteed using vanilla generative large language models (Sun et al., 2023; Roy et al., 2024). This is the case because the validity of tokens w.r.t. those constraints is not reflected in the standard unconstrained greedy or beam search decoding approaches. Typically, the output is determined by how likely the model considers specific tokens to be in specific positions. However, these predictions can be sometimes wrong or violate output constraints, in the worst case rendering the entire output to be invalid. This is especially relevant when the output needs to be parsed or executed, like when generating code or formulas, e.g., SMT formulas (Pan et al., 2023; Sun et al., 2023) or SQL queries (Scholak et al., 2021; Lin et al., 2020). In those domains, “almost correct” is usually equivalent to invalid and wrong, as those outputs are then commonly simply rejected by the parsers or execution engines after generation. Related work like Sun et al. (2023); Roy et al. (2024) also shows that this is an actual problem and that validity rates even of the most recent language models are far from perfect for applications where it is important to strictly follow a certain grammar.

It is thus an important goal to ensure that the output of a model follows a certain semantic and syntactic structure. As one solution we can consider grammar-constrained decoding approaches that ensure that the decoded structure follows the (production) rules of a given (semantic) grammar. In this paper, we thus experimentally investigate the impact of a grammar-constrained decoding approach on the well-formedness and correctness of the output of structured information extraction models. We investigate this impact in the context of fine-tuned large language models that rely on a supervised setting to adapt the model parameters by optimizing parameters on the basis of a given labeled dataset. Further, our focus is on what we call *low-resource settings* by which we denote settings in which at most 500 training examples are available (compare Roy et al., 2024) and where the number of model parameters is less than 500 million parameters. The first restriction matches typical information extraction settings which rely on human-labeled text examples that are costly to obtain. The second restriction corresponds to situations where models are trained on standard hardware.

The low-resource setting we consider in this paper is of practical relevance. First, the training of large models requires substantial energy resources and generates a corresponding carbon footprint (Strubell et al., 2019; George et al., 2023), such that reducing energy consumption by models with a smaller footprint is an important goal. Second, in many settings, pre-trained models are used in zero-shot or few-shot settings, but they are not fine-tuned to a specific problem due to the large costs and resources needed for that. Nevertheless, in order to produce domain-adapted performance, it is important to optimize models on the actual target task, so fine-tuning is still an important paradigm. Yet, when fine-tuning models on a particular task, it remains a larger challenge to manually annotate thousands of examples. In many cases, resources available for annotating data for research tasks are limited. Especially in the biomedical domain as we consider in this paper, requiring scarcely available domain expertise for the annotation of texts, it is rare to find datasets with several thousands of annotated documents. For instance, the well-known Genia corpus (Kim et al., 2003) features 1, 999 annotated abstracts. Biomedical named-entity recognition and entity linking corpora like MedMentions (Mohan and Li, 2019) contain around 4, 000 abstracts, and biomedical text summarization datasets like MeQSum (Abacha and Demner-Fushman, 2019) comprise 1, 000 summarized health questions. Additionally, the BLUE benchmark (Peng et al., 2019) contains corpora with different sizes, ranging from 64 to 11, 232 examples. However, the second largest dataset with 5, 203 already contains considerably fewer examples. All in all, biomedical datasets in general and more specialized clinical datasets in particular tend to be comparably small.

Taken together, the considered low-resource setting assuming models to be in the order of hundreds of millions of parameters and hundreds of training examples is of practical value and relevance. In this setting, we empirically investigate the impact of a domain-specific grammar that is used at decoding time to ensure that output structures meet well-formedness criteria.

In the slot-filling information extraction paradigm we consider, where a template structure must be filled with slots extracted from the text, an important constraint is that the elements of the slots actually come from the original text. In fact, when using generative models, there is the risk that the model “hallucinates” slot fillers that were never mentioned in the text. Thus, in addition to considering decoding following a domain-specific grammar, we also consider the impact of pointer generators that additionally use the attention to the input tokens at each output step and thus allow a model to “copy” from the input more directly.

Given this motivation, in this article, we pose three research questions:

**RQ1**. Impact of grammar-constrained decoding: How does grammar-constrained decoding (GCD) affect the performance of fine-tuned large language models in low-resource settings compared to greedy decoding (noGCD) w.r.t. structured information extraction tasks?
**RQ2**. Combining grammar-constrained decoding with pointer generators: Does the combination of grammar-constrained decoding with pointer generators improve results?
**RQ3**. Performance of different attention aggregation strategies: Which attention aggregation method (ptr-sum/ptr-max) works best for pointer generators combined with grammar-constrained decoding?

The specific application domain we consider in our paper is structured information extraction in the clinical trial domain. In particular, we focus on the extraction of PICO-related information from abstracts describing the results of randomized clinical trials (RCTs). Hereby, PICO refers to Patient, Intervention, Comparison and Outcomes, representing the key concepts relevant in describing the results of a randomized clinical trial (Schardt et al., 2007; Richardson et al., 1995).

Taken together, to the best of our knowledge, our work contributes novel insights w.r.t. the benefits and drawbacks of using grammar-constrained decoding and pointer generators with fine-tuned generative large language models in low-resource settings. Our paper features the following contributions:

- We show the positive impact of grammar-constrained decoding on generative LLMs fine-tuned for structured information extraction in a low-resource setting, improving *F*_1_ scores from 0.062 to 0.413 and from 0.102 to 0.47 for type 2 diabetes and glaucoma datasets, respectively.
- We show that adding pointer generators on top of grammar-constrained decoding has a negative impact on the performance, decreasing *F*_1_ scores from 0.413 to 0.263 and from 0.47 to 0.292 for type 2 diabetes and glaucoma datasets, respectively.
- We investigate the influence of different attention aggregation strategies (determining what to do with the attention values if a token occurs multiple times in the input) on the performance of pointer generators, considering the sum and the maximum function in particular. We show that the choice depends on the base model, as the maximum function generates the overall best results in this category, but only if paired with the led-base-16384 model, whereas it yields the worst results when used together with the flan-t5-base. In contrast, the sum function achieves comparable although not the best scores for both tested base models.
- An ablation experiment with a larger model analyzes the influence of model size on the benefits reached via grammar-constrained decoding, showing that the performance improvements persist or even increase when using larger models, suggesting that the model size alone does not solve the problem of LLMs not always sticking to the desired output specification.

In the following, we first discuss how this paper is embedded into related work (Section 2) before discussing the methods presented in this paper and modifications applied to the base models (Section 3). Afterwards, the conducted experiments and used datasets are described in detail in Section 4. The results of those experiments are then reported in Section 5 and discussed w.r.t. the research questions in the following Section 6. Finally, we conclude our findings with a conclusion (Section 7).

## 2 Related work

Many natural language processing tasks require structured output, including event extraction (Lu et al., 2021), syntactic and semantic parsing (Roy et al., 2024), symbolic expression generation like SMT formulas (Pan et al., 2023; Sun et al., 2023) or generating SQL queries (Scholak et al., 2021; Lin et al., 2020), which have a clearly defined syntax. Additionally, except for Lin et al. (2020), pointer generators are rarely evaluated in related work whereas this is a main focus of our work.

Because of the typically strict constraints on the output structure, various types of constrained decoding algorithms have evolved over the years, e.g., by pruning invalid tokens in beam search algorithms (Anderson et al., 2017), incremental parsing techniques (Scholak et al., 2021) or trie-based constraints (Cao et al., 2021; Lu et al., 2021). These kinds of constraints, however, are different in multiple ways from the flexible generalized grammar-based approach that is pursued in this paper. For example, the trie-based constrained decoding for event extraction proposed by Lu et al. (2021) is used to generate trie-like structures to capture event structures present in a given text with generative models. The structural properties are, however, not generalized to the level where constraints of the desired structure can be flexibly formulated as grammar rules. Nevertheless, the approach and the trie-like structure closely resemble parse trees, such that the approach is a specific instance of the more general approach that we examine in this paper, relying on context-free grammars to guide decoding.

Along these lines, recent work by Geng et al. (2023) has examined the impact of using a context-free grammar and Grammatical Framework (Ranta, 2019) for constrained decoding, aiming to provide a unified approach to address various kinds of structures required in different domains and tasks. While they have focused on pre-trained models, our work specifically focuses on investigating the impact of grammar-constrained decoding in fine-tuning settings.

The effect of constrained decoding has been evaluated with respect to fine-tuned models on tasks other than information extraction, that is on the task of generating SQL queries (Scholak et al., 2021). Stengel-Eskin et al. (2024) have presented an approach to convert ambiguous natural language descriptions into logic formulas and code, but considering zero- and few-shot settings instead of fine-tuning settings as considered in our work.

While Pan et al. (2023) do not use constrained decoding, they instead explore the effect of self-refinement in case of symbolic reasoner parsing errors on the validity of the generated logic formulas. Roy et al. (2024) propose a benchmark consisting of syntactic and semantic parsing tasks and evaluate it on a range of models, both fine-tuned and non-fine-tuned models as well as with and without grammar constraints. Compared to this work, neither the clinical domain nor structured information extraction are in the focus of their evaluations nor are pointer generators considered as a supporting mechanism in detail.

In conclusion, our work follows the lines of Geng et al. (2023), Lu et al. (2021), and Roy et al. (2024) by testing the benefit of grammar-constrained decoding in NLP settings. In contrast to previous work, we investigate the impact of grammar-constrained decoding in a fine-tuning setting and in particular on the task of structured information extraction, focusing on low-resource settings. The impact of grammar-constrained decoding has not been investigated in low-resource settings before.

With respect to the biomedical and clinical domain, various approaches have been proposed for tasks like relation extraction (Jiang and Kavuluru, 2023; Kim and Meystre, 2020), question answering (Wang et al., 2020), named entity recognition (Stylianou et al., 2021) or event extraction (Wang et al., 2020; Ramponi et al., 2020; Zhu and Zheng, 2020; Huang et al., 2020; Trieu et al., 2020). Some approaches and models even aim to detect and extract information from (randomized) clinical trial abstracts, e.g., slot fillers (Papanikolaou et al., 2022) or clinical trial outcomes (Abaho et al., 2022b,a, 2021; Ganguly et al., 2021). Taken together, all of the listed examples either deal with a different task, do not work in a sequence-to-sequence manner as our approach does, or lack the nested structure and dependencies of Patient, Intervention, Comparison, Outcomes (PICO) templates and slots that are dealt with in this paper.

Considering the latter, this work represents randomized controlled trials (RCTs) in a structured way using the already mentioned Patient, Intervention, Comparison, Outcomes (PICO) framework (Schardt et al., 2007; Richardson et al., 1995). This framework consists of templates with corresponding slots, which can be filled with either textual data or again with template instances. In contrast to our approach, most related work like Schmidt et al. (2020) and Zhang et al. (2020) treats PICO elements as flat classes, i.e., parts of sentences which are just labeled, e.g., P or I. In contrast, our approach treats PICO elements as nested structures in order to do justice to the complex information that is presented in those elements. In particular, we structure the information by means of templates with slots that have to be filled with some portion of text or other template instances, thus creating a nested structured representation of the PICO information. Furthermore, there are also some approaches (Whitton and Hunter, 2023; Dhrangadhariya et al., 2021) which aim to generate more structured representations of the PICO information in RCT abstracts, but differ in terms of architecture and decoding approaches additionally to the structures generated still being less complex than the recursive template structure we use in this paper.

## 3 Methods

**Figure 1:**
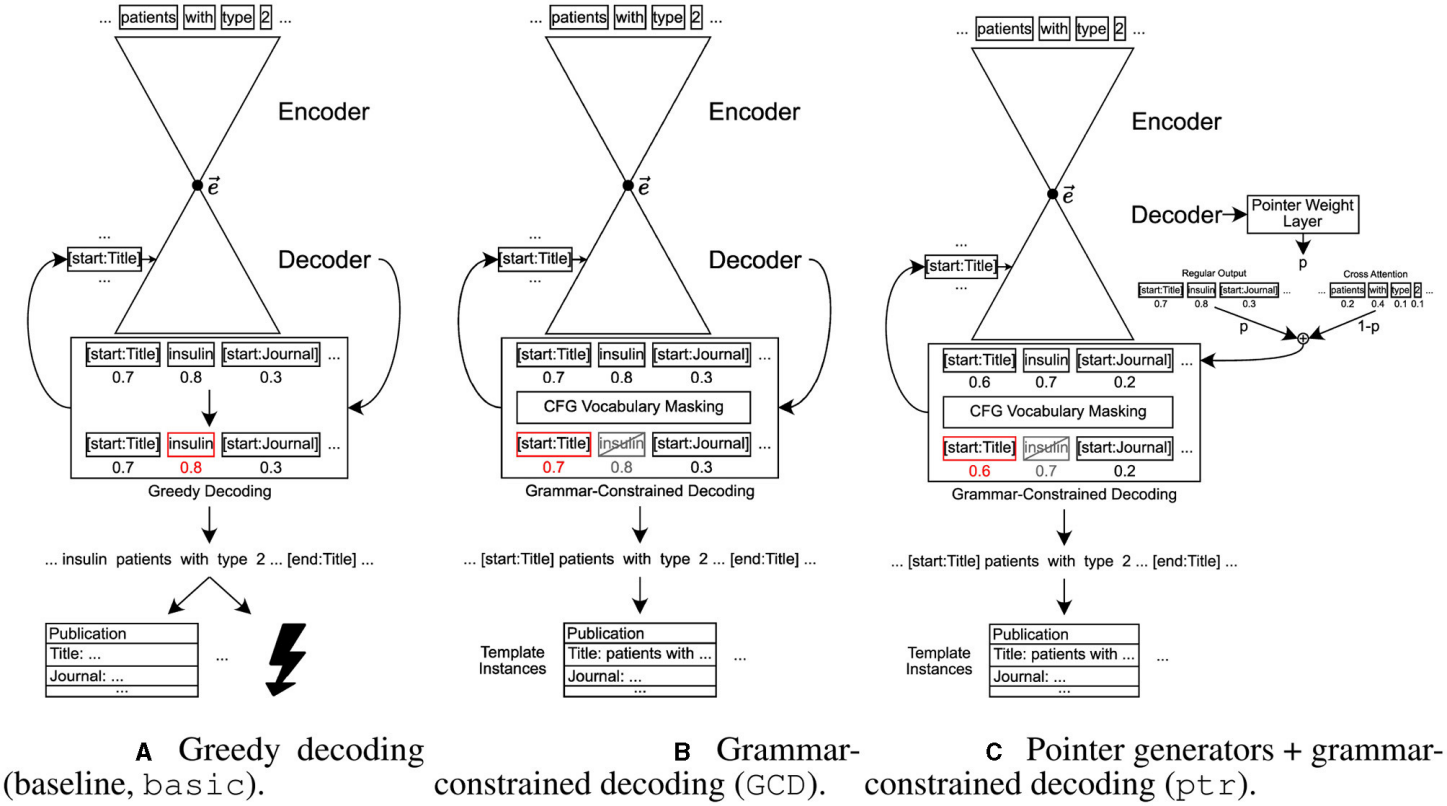
Illustration of the baseline model as well as the two adjustments added to that baseline, grammar-constrained decoding and pointer generator-like behavior. Words in boxes represent single tokens, numbers below those boxes symbolize outputs from the decoder, where higher values stand for a higher probability that this is the best next token as estimated by the model. For greedy decoding, the token with the highest value is chosen. For GCD, a filter is applied before, visualized as gray, crossed-out boxes for tokens that are filtered out. Red boxes show the selected token. **(A)** Greedy decoding (baseline, basic). **(B)** Grammar-constrained decoding (GCD). **(C)** Pointer generators + grammar-constrained decoding (ptr).

In this section, we describe how we approach the structured information extraction task and describe two aspects that we add to the “raw” sequence-to-sequence model, namely grammar-constrained decoding and pointer generator-like behavior. This is also illustrated in Figure 1.

### 3.1 Task

**Figure 2:**
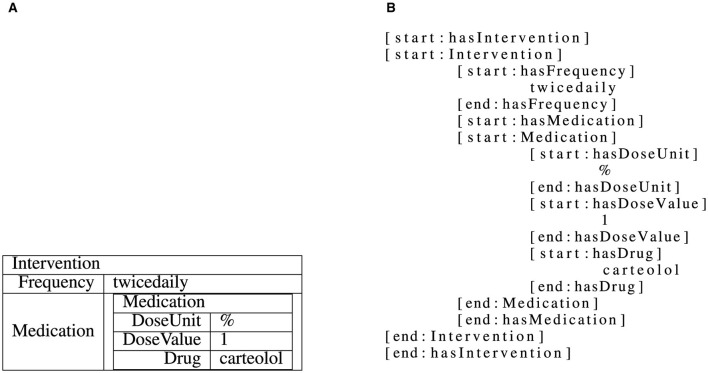
Illustration of a linearized intervention template instance (Witte et al., 2024). The nested template instance as shown in **(A)** is linearized to a flat string as shown in **(B)**, adding start and end tokens for both textual and complex, nested slots.

In this paper, we tackle the task of structured information extraction from RCT abstracts. We do this in a sequence-to-sequence manner by providing an abstract as input and expecting structured results in terms of the C-TrO ontology (Sanchez-Graillet et al., 2019) as an output. The information extraction task is framed as a slot-filling approach in this paper. In such a task, the templates, i.e., collections of slots, which are defined by the C-TrO ontology, need to be filled using text from an RCT abstract. A slot can be filled with one of two types of slot-fillers, with the type depending on which slot of a template is filled: text from the RCT abstract or a (nested) instance of another template. The grammar used to represent and linearize the different parts of the C-TrO ontology is the one from (Witte et al., 2024). An example is illustrated in Figure 2.

### 3.2 Baseline

As a baseline, the dataset comprising pairs of an abstract and the corresponding linearized C-TrO ontology representation is used to fine-tune a sequence-to-sequence model for the specific task. For this purpose, a encoder-decoder model is fine-tuned “as-is” without any of the modifications presented in the remainder of this section. The baseline will also be called basic in the following.

In order to formally define the decoding methods as well as the pointer generator-like behavior later, we first have to define some general notation for vectors and matrices and how to access their values. This notation is inspired by the way NumPy (Harris et al., 2020) arrays are accessed.

Let $\vec{v}\in {\mathbb{R}}^{d}$ be a *d*-dimensional vector the elements of which are accessed using square brackets, i.e., $\vec{v}\left[i\right]$ with 0 ≤ *i* ∈ ℕ < *d* to retrieve the *i*-th element of $\vec{v}$. Similarly, let $M\in {\mathbb{R}}^{d_{1}\times \cdots \times d_{n}}$ be an *n*-dimensional matrix with *d*_*i*_ ∈ ℕ values in each dimension 1 ≤ *i* ∈ ℕ ≤ *n*. In order to access a single element of the matrix, an index for every dimension of *M* has to be given via bracket notation: *M*[*j*_0_, …, *j*_*n*_] with 0 ≤ *j*_*k*_ ∈ ℕ < *d*_*k*_ for every 1 ≤ *k* ∈ ℕ ≤ *n*. To access larger parts of a matrix, : can be used instead of an index to indicate that all values in that dimension are selected instead of just a single element of it. With this notation in mind, we can now define the used decoding algorithms in the following.

For the baseline, we use an unconstrained greedy decoding, called noGCD, which always chooses the token with the highest corresponding value from the final distribution regardless of any constraints. Let *dist* be the final token distribution created by the model, then this means:


(1) $$\begin{array}{l} nextToken=\underset{i}{\mathrm{argmax}}dist\left[i\right] \end{array}$$

### 3.3 Grammar-constrained decoding

In the grammar-constrained decoding approach we rely on a decoding approach that ensures that the production rules of a given context-free grammar are enforced. For decoding, we thus use a grammar-constrained decoding algorithm, denoted as GCD, which masks the vocabulary in every step based on the possible next tokens determined by the given context-free grammar.

Concretely, this means the following: Let *accepted* be the set of tokens which are valid according to the context-free grammar used by the decoding algorithm and represented by their token id, i.e., their index in the tokenizer vocabulary 𝕍. Then the vocabulary or distribution mask is defined as follows:


(2) $$\begin{array}{l} mask\in {\left(\mathbb{R}\cup \left\{-\infty \right\}\right)}^{\left|𝕍\right|}mask\left[i\right]=\{\begin{array}{cc} 0 & \text{if }i\in accepted\\ -\infty & \text{otherwise} \end{array} \end{array}$$

The next token is then determined using the masked token distribution:


(3) $$\begin{array}{l} nextToken=\underset{i}{\mathrm{argmax}}\left(dist\left[i\right]+mask\left[i\right]\right) \end{array}$$

In practice, this is implemented using the Lark parsing toolkit (Shinan, 2024) together with the core grammar which can be found in Supplementary material. The decoding phase consists of two parts. First, the model output sequence is generated using grammar-constrained decoding as described above. As a second step, the generated output sequence is parsed, returning a parse tree which is then further processed to build template instances from that parse tree, which can then be evaluated.

The first decoding phase is implemented by utilizing the Lark interactive parser, which is available when using the LALR parsing mode (DeRemer, 1969). The next possible tokens corresponding to the look-ahead become accessible and can be utilized to create the token distribution mask described above. As no backtracking mechanism is currently implemented in the decoding algorithm, the used grammar needs to be defined in a way which allows to unambiguously decide for the correct token in a single step, as reverting a previous decision based on later tokens is currently not possible. After decoding, the regular parsing mode can then be used to create an actual parse tree from the generated output.

In our structured information extraction task and in order to keep the decoding process as efficient as possible, the first decoding phase using the interactive parser features the core grammar with a simple definition of the free-text non-terminal POINT. This definition only avoids matches of [start: and [end: in order to prevent errors related to the special tokens indicating boundaries of slots and templates in the linearization. As this decoding process can by construction only generate valid tokens in each step, no further validation is necessary here.

In contrast, the second phase, which can also be used separately to parse stored linearizations in order to reconstruct the corresponding template instances, uses a more restrictive definition of the free text non-terminal POINT. In this phase, the definition of POINT is constructed from the tokenizer vocabulary in such a way that greedy matching is applied in case there are multiple possible tokenizations for a string. Considering the typically thousands of tokens in a tokenizer vocabulary, this increases the size of the resulting grammar substantially, but in exchange ensures a meaningful parse tree even for different tokenizers.

### 3.4 Pointer generators

The second adjustment made to the baseline method is adding pointer generator-like behavior. Therefore, this category of models will be called ptr in the following, or more precisely ptr-max when using the maximum function and ptr-sum when using the sum function for aggregation of the attention values when some token occurs multiple times in the input. Adding pointer generator-like behavior is intended to help the model copy tokens from the input, which is an important part of the considered information extraction task.

More concretely, we add a linear layer followed by a sigmoid activation as well as a slightly different method to calculate the final distribution over the token vocabulary. The method works in a similar fashion to the pointer generator described by See et al. (2017) and Deaton et al. (2019), but relying on a different architecture described in more detail below.

For this purpose, we define the calculation of the token distribution for ptr models as follows: Let *l* be the latest prediction logits in a generation step (omitting the dimension for batching) of the generative model and *dist*_*gen*_ = *softmax*(*l*) the corresponding classical generative token distribution. Moreover, let *p*_*gen*_ ∈ [0, 1] be the output of an additional linear layer with sigmoid activation applied to it afterwards. *p*_*gen*_ is the fraction to which the classical token distribution influences the final token distribution. The pointer distribution will instead be multiplied with 1−*p*_*gen*_.

Now, let *C* ∈ ℝ^*heads*×*inputTokens*^ be the normalized (i.e., the sum of all values of a head is 1) values of the last cross-attention layer of the latest generation step with *heads* attention heads and *inputTokens* input tokens. We then first calculate the mean over all attention heads, i.e., $\vec{c}=\frac{1}{heads}\overset{heads-1}{\underset{i=0}{\sum }}C\left[i,:\right]$.

$\vec{c}$ is, however, a distribution over the input tokens and not the tokenizer vocabulary 𝕍 unlike *dist*_*gen*_. To transform $\vec{c}$ into a suitable distribution over 𝕍, on the one hand we have to decide what happens when there are multiple attention values for a token because it occurs multiple times and on the other hand aggregate those values at the position of the correct token in 𝕍 in some resulting distribution.

In this work, we evaluated both the maximum (ptr-max) and the sum operation (ptr-sum). In the case of ptr-max we use the maximum operation to determine the maximum attention value for a given token $t\in \vec{c}$ to be used in the pointer distribution at the corresponding position (see Equation 4). Correspondingly, in case of ptr-sum, the sum of all attention values of a token *t* is used in the pointer distribution (see Equation 5).

The resulting pointer token distribution *dist*_*ptr*_ is therefore determined as follows:


(4) $$\begin{array}{l} {dist}_{ptr}^{max}\left[i\right]:=\underset{0\leq j<inputTokens}{\max}\left\{\vec{c}\;\left[j\right]|\vec{input}\left[j\right]=i\right\}\\ {dist}_{ptr}\in {\mathbb{R}}^{\left|𝕍\right|},0\leq i\in \mathbb{N}<\left|𝕍\right| \end{array}$$

(5) $$\begin{array}{l} {dist}_{ptr}^{sum}\left[i\right]:=\underset{0\leq j<inputTokens}{\sum }\left\{\vec{c}\;\left[j\right]|\vec{input}\left[j\right]=i\right\}\\ {dist}_{ptr}\in {\mathbb{R}}^{\left|𝕍\right|},0\leq i\in \mathbb{N}<\left|𝕍\right| \end{array}$$

with $\vec{input}\in {\mathbb{N}}^{inputTokens}$ being the vector of input token ids.

The final token distribution used for decoding in case of the ptr-max model is then defined as follows:


(6) $$\begin{array}{ll} {dist}_{final}^{max}=p_{gen}\cdot {dist}_{gen}+\left(1-p_{gen}\right)\cdot {dist}_{ptr}^{max} & {dist}_{final}^{max}\in {\mathbb{R}}^{\left|𝕍\right|} \end{array}$$

Similarly, the final token distribution for ptr-sum is:


(7) $$\begin{array}{ll} {dist}_{final}^{sum}=p_{gen}\cdot {dist}_{gen}+\left(1-p_{gen}\right)\cdot {dist}_{ptr}^{sum} & {dist}_{final}^{sum}\in {\mathbb{R}}^{\left|𝕍\right|} \end{array}$$

These distributions over the tokenizer vocabulary 𝕍 are then used analogously in the decoding process to generate a sequence of output tokens.

## 4 Experiments

In this section, the different experiments conducted in this paper are described, together with the dataset and other relevant experimental settings used for training and evaluation.

### 4.1 Dataset

In our experiments, we reuse the dataset provided by Witte and Cimiano (2022) and Witte et al. (2024), which consists of abstracts of RCTs about type 2 diabetes and glaucoma and annotated according to the C-TrO ontology (Sanchez-Graillet et al., 2019). The dataset comprises a total of 211 documents, 104 on type 2 diabetes and 107 on glaucoma. The 104 type 2 diabetes documents are split up into training, validation and test sets of size 68, 16, and 20, respectively. Analogously, the 107 glaucoma documents are split up into training, validation and test sets of size 69, 17, and 21, respectively. Thus, we use the same dataset as well as the same fixed train-validation-test split as Witte and Cimiano (2022) and Witte et al. (2024) and run separate experiments for those two diseases. The exact corresponding number of tokens certainly varies with the used model, tokenizer and pre-processing steps. However, to give a rough estimate, the whole dataset, including both input and output tokens, consists of around 300K tokens in total, with ~200K training tokens, 50K validation tokens and around 60K test tokens. These numbers are roughly split in half by disease, i.e., around 100K training tokens for type 2 diabetes and glaucoma each. With these sizes, the used dataset can be considered small when compared to typical fine-tuning tasks explored in related work, especially considering the complexity of the task and length of the targeted output (e.g., even datasets with more samples are still considered low-resource in Roy et al., 2024). In contrast, the dataset is also much larger than the data that is provided to large language models in zero- to few-shot prompting settings (e.g., in Stengel-Eskin et al., 2024) and this way provides an interesting perspective on constrained decoding used in combination with fine-tuning in low-resource environments.

### 4.2 Models

In our experiments, we tested two different encoder-decoder transformers (Vaswani et al., 2017) as base models, namely google/flan-t5-base^1^ (Chung et al., 2022, 223M parameters) and allenai/led-base-16384^2^ (Beltagy et al., 2020, 161M parameters). These base models were then evaluated in four variants:

1. basic: Vanilla model without modifications, paired with standard greedy decoding (noGCD).
2. GCD: Vanilla model without modifications, paired with grammar-constrained decoding.
3. ptr: Model with additional layers for pointer generator-like behavior, paired with grammar-constrained decoding, using different attention aggregation functions for the case of multiple occurrences of a token in the input sequence:
  (a) ptr-max: Using the maximum function for aggregating attention values of multiple token occurrences.
  (b) ptr-sum: Using the sum function for aggregating attention values of multiple token occurrences.

Therefore, when adding pointer generator-like behavior, exclusively grammar-constrained decoding is considered. The used decoding approach itself is then identical to the grammar-constrained decoding described in Section 3.3. However, in this case ${dist}_{final}^{max}$ and ${dist}_{final}^{sum}$ are used for ptr-max and ptr-sum, respectively, as token distributions over the respective vocabulary instead of the classical distribution *dist*.

### 4.3 Experimental setup

For the considered models and diseases, we ran hyperparameter optimizations using Optuna (Akiba et al., 2019) with 30 trials each and measuring performance with grammar-constrained decoding using validation *F*_1_ scores, calculated as described in Section 4.4. The noGCD values are not calculated on models that were trained separately, but instead the already trained models are additionally evaluated with a different decoding technique. The training procedure itself is the same and GCD behaves just like standard greedy decoding when valid output sequences are generated, so that the difference should not be relevant. However, there could be other training parameters which are more beneficial for noGCD than for GCD (which was used to measure validation performance), but this has not been evaluated in this work.

In each executed trial, a λ for the lambda learning rate scheduler (between 0.9 and 1.0, using logarithmic domain, learning rate calculated with *lr*(*epoch*) = λ^*epoch*^) as well as a corresponding initial learning rate (between 1*e*^−3^ and 1*e*^−5^, using logarithmic domain) are sampled from Optuna. The chosen batch size is 1 and the number of epochs is 50 in all experiments, each of which is then executed on a single NVIDIA A40 GPU.

The best hyperparameters for each disease-model-setting-combination are then used to train 10 additional models. Unless stated differently, mean and standard deviation in tables refer to the different results of these 10 training runs. The means and standard deviations of the test *F*_1_ scores of these 10 trained models are listed in Table 1 for the experimental setting of 1 and in Table 2 for 1 and 1.

**Table 1:** Evaluation of the impact of grammar-constrained decoding vs. greedy decoding (1).

↓**Setting**		**Dataset→**	**Type 2 diabetes**	**Glaucoma**
**Model**	**Type**	**Decoding**	**Mean** *F*_1_ **(**±σ**)**	**Mean** *F*_1_ **(**±σ**)**
flan-t5-base	Basic	GCD	**0.413 (±0.13)**	**0.47 (±0.061)**
flan-t5-base	Basic	noGCD	0.062 (±0.041)	0.045 (±0.043)
led-base-16384	Basic	GCD	0.301 (±0.102)	0.292 (±0.12)
led-base-16384	Basic	noGCD	0.016 (±0.029)	0.102 (±0.049)

Mean and standard deviation σ of test *F*_1_ scores across 10 models trained using best-performing (*F*_1_ on validation dataset) configuration found in 30 trials of hyperparameter optimization. Numbers rounded to three decimal places, best configuration of each disease marked bold.

**Table 2:** Evaluation of the impact of pointer generators and the used attention aggregation method (1 and 1).

↓**Setting**		**Dataset→**	**Type 2 diabetes**	**Glaucoma**
**Model**	**Type**	**Decoding**	**Mean** *F*_1_ **(**±σ**)**	**Mean** *F*_1_ **(**±σ**)**
flan-t5-base	basic	GCD	**0.413 (±0.13)**	**0.47 (±0.061)**
flan-t5-base	ptr-max	GCD	0.092 (±0.075)	0.091 (±0.015)
flan-t5-base	ptr-sum	GCD	0.16 (±0.074)	0.211(±0.084)
led-base-16384	ptr-max	GCD	0.263 (±0.067)	0.272 (±0.046)
led-base-16384	ptr-sum	GCD	0.236 (±0.064)	0.216 (±0.078)

Mean and standard deviation σ of test *F*_1_ scores across 10 models trained using best-performing (*F*_1_ on validation dataset) configuration found in 30 trials of hyperparameter optimization. Numbers rounded to three decimal places, best configuration of each disease marked bold.

However, as the dataset consists of information that is stored in a complex nested template structure, it is not immediately possible to train a model on this data. Therefore, the structure is linearized similarly to XML, i.e., with start [start:<*slot or template name*>] and end [end:<*slot or template name*>] tags for slots and templates, which allows to freely nest even templates in other templates. For each of these tags, special tokens are added to the vocabulary. In order to reduce the input data variance and allow the models to learn the relations more easily, an (arbitrary but fixed, e.g., alphanumerically sorted) order is enforced when linearizing templates and slots. An example for a linearized nested template with both textual slots and a slot which contains a template is given in Figure 2.

In order to answer our research questions we focus on the following two experimental settings:

- Impact of grammar-constrained decoding (1): We compare the setting in which a grammar is used to constrain the decoding (GCD) and the case in which it is not used (noGCD), i.e., in which standard greedy decoding is applied.
- Impact of pointer generators and attention aggregation methods (1 and 1): We quantify the impact of adding pointer generator-like behavior, comparing two different attention aggregation methods (sum/maximum) for the GCD case.

### 4.4 Evaluation

Evaluating the predicted templates against the ground truth templates is again not a trivial task, as, in some cases, various template instances have to be aligned to each other. This is done by optimizing the *F*_1_ score across all possible alignments/matchings by modeling it as a linear inequality system and maximizing for the resulting *F*_1_ score. The *F*_1_ score for a single predicted and ground truth template is calculated by first determining true positives, false positives and false negatives for the textual slot fillers of the two templates.

Two textual slot fillers are considered equal when the concatenation of the tokens of that slot filler has a similarity of ≥0.9 according to the following normalized Levenshtein similarity measure:


(8) $$\begin{array}{cc} normLevenshteinSim\left(s_{1},s_{2}\right) & :=1-\frac{levenshteinDistance\left(s_{1},s_{2}\right)}{\max\left(\left|s_{1}\right|,\left|s_{2}\right|\right)} \end{array}$$

where *levenshteinDistance* in the above definition refers to the Levenshtein distance proposed by Levenshtein (1966). The concatenation and Levenshtein similarity calculation step is necessary to avoid problems regarding the tokenization, e.g., situations where the generated text is equal but the tokenization is slightly different and leading to low scores otherwise. Furthermore, this reduces the bias toward long textual slots with many tokens like the Title slot of a publication which is easy to predict and typically consists of many tokens compared to, e.g., Outcome template instances comprising primarily numbers and short units in most cases. We consider this to be a more meaningful and fair evaluation, albeit having the drawback of our results not being directly comparable to those reported by Witte et al. (2024) and Witte and Cimiano (2022).

Correspondingly, slot fillers which are equal w.r.t. the above definition and occur in both templates are counted as a true positive, those which only occur in the predicted template are counted as a false positive, and those which only occur in the ground truth template are counted as a false negative. Moreover, identical and different template slot fillers are added to those numbers in the same fashion, but without applying the approach recursively, i.e., only completely identical template slot fillers are considered equal and counted as one accordingly. From the sum of these true positives, false positives and false negatives then the *F*_1_ score of a template is calculated.

## 5 Results

The results of the conducted experiments relevant for 1 can be found in Table 1 and for 1 as well as 1 in Table 2. In addition to the overall performance scores presented in Tables 1, 2, the mean scores per template are shown in Table 3 as well as per slot in Table 4. In both cases, the values for the glaucoma dataset for the GCD models are listed there as an example. The remaining data can be found in Supplementary material. This section briefly mentions the most important results w.r.t. all three research questions together with a small ablation study w.r.t. the model size.

**Table 3:** Glaucoma test *F*_1_ scores per template.

	**Glaucoma** ***F***_**1**_		
**Template name**	**Basic GCD**	**ptr-max GCD**	**ptr-sum GCD**
Arm	0.21 (±0.08)	0.07 (±0.06)	0.05 (±0.07)
ClinicalTrial	0.53 (±0.05)	0.3 (±0.07)	0.22 (±0.09)
DiffBetweenGroups	0.15 (±0.08)	0.07 (±0.04)	0.04 (±0.03)
Endpoint	0.33 (±0.06)	0.22 (±0.03)	0.19 (±0.07)
Intervention	0.49 (±0.1)	0.23 (±0.07)	0.21 (±0.1)
Medication	0.51 (±0.11)	0.3 (±0.06)	0.27 (±0.12)
Outcome	0.26 (±0.05)	0.1 (±0.04)	0.09 (±0.05)
Population	0.47 (±0.06)	0.25 (±0.08)	0.16 (±0.1)
Publication	0.69 (±0.06)	0.34 (±0.1)	0.0 (±0.0)

Mean and standard deviation σ per template of glaucoma test *F*_1_ scores, considering the best-performing base model of each category. Numbers rounded to two decimal places.

**Table 4:** Glaucoma test *F*_1_ scores per slot.

	**Glaucoma** ***F***_**1**_		
**Slot name**	**Basic GCD**	**ptr-max GCD**	**ptr-sum GCD**
AggregationMethod	0.52 (±0.08)	0.34 (±0.07)	0.31 (±0.13)
AnalysesHealthCondition	0.87 (±0.02)	0.59 (±0.05)	0.57 (±0.1)
Author	0.61 (±0.04)	0.34 (±0.11)	0.21 (±0.12)
BaselineUnit	0.55 (±0.06)	0.38 (±0.04)	0.3 (±0.1)
BaselineValue	0.47 (±0.17)	0.18 (±0.1)	**0.19 (±0.16)**
CTDesign	0.63 (±0.05)	0.39 (±0.09)	0.21 (±0.13)
CTduration	0.68 (±0.09)	0.32 (±0.1)	0.25 (±0.14)
ChangeValue	0.43 (±0.07)	0.21 (±0.05)	0.21 (±0.09)
ConclusionComment	0.59 (±0.06)	0.25 (±0.1)	0.14 (±0.09)
ConfIntervalDiff	0.11 (±0.14)	0.02 (±0.06)	0.0 (±0.0)
Country	0.76 (±0.1)	0.39 (±0.12)	0.25 (±0.14)
DeliveryMethod	0.23 (±0.2)	0.05 (±0.11)	**0.11 (±0.14)**
DiffGroupAbsValue	0.11 (±0.13)	0.04 (±0.09)	0.02 (±0.06)
DoseUnit	0.69 (±0.13)	0.51 (±0.11)	0.45 (±0.2)
DoseValue	0.65 (±0.12)	0.36 (±0.05)	0.31 (±0.14)
Drug	0.45 (±0.07)	0.29 (±0.07)	0.23 (±0.1)
EndoPointDescription	0.21 (±0.06)	0.16 (±0.04)	0.16 (±0.06)
FinalNumPatientsArm	0.03 (±0.11)	0.0 (±0.0)	0.0 (±0.0)
FinalNumberPatientsCT	0.11 (±0.16)	0.05 (±0.11)	0.0 (±0.0)
Frequency	0.67 (±0.06)	0.4 (±0.08)	0.37 (±0.12)
Journal	0.66 (±0.07)	0.36 (±0.1)	0.24 (±0.16)
MeasurementDevice	0.06 (±0.13)	0.0 (±0.0)	0.0 (±0.0)
NumberAffected	0.37 (±0.27)	0.01 (±0.02)	**0.03 (±0.06)**
NumberPatientsArm	0.38 (±0.2)	0.13 (±0.13)	0.1 (±0.15)
NumberPatientsCT	0.48 (±0.07)	0.27 (±0.19)	0.23 (±0.16)
ObjectiveDescription	0.36 (±0.09)	0.24 (±0.06)	0.12 (±0.1)
ObservedResult	0.01 (±0.02)	**0.02 (±0.02)**	0.01 (±0.02)
PMID	0.76 (±0.07)	0.32 (±0.12)	0.21 (±0.14)
PValueChangeValue	0.01 (±0.04)	**0.08 (±0.13)**	0.03 (±0.07)
PercentageAffected	0.19 (±0.1)	0.06 (±0.05)	0.05 (±0.07)
Precondition	0.18 (±0.06)	0.12 (±0.06)	0.06 (±0.07)
PublicationYear	0.88 (±0.09)	0.36 (±0.12)	0.29 (±0.18)
PvalueDiff	0.24 (±0.04)	0.14 (±0.06)	0.09 (±0.05)
RelativeChangeValue	0.1 (±0.18)	0.05 (±0.08)	**0.1 (±0.15)**
RelativeFreqTime	0.31 (±0.17)	0.07 (±0.12)	0.05 (±0.16)
ResultMeasuredValue	0.39 (±0.11)	0.16 (±0.09)	0.11 (±0.08)
SdDevBL	0.31 (±0.14)	0.07 (±0.08)	0.07 (±0.07)
SdDevChangeValue	0.24 (±0.09)	0.08 (±0.07)	**0.09 (±0.12)**
SdDevResValue	0.43 (±0.12)	0.15 (±0.1)	0.14 (±0.11)
SdErrorChangeValue	0.11 (±0.18)	0.03 (±0.11)	0.0 (±0.0)
TimePoint	0.36 (±0.05)	0.18 (±0.1)	0.14 (±0.1)
Title	0.56 (±0.05)	0.32 (±0.1)	0.21 (±0.14)
Total Micro *F*_1_ Score	0.47 (±0.06)	0.27 (±0.05)	0.22 (±0.08)

Mean and standard deviation σ per slot of glaucoma test *F*_1_ scores, considering the best-performing base model of each category. Numbers rounded to two decimal places. Notable exceptions where either ptr-max outperforms basic or ptr-sum outperforms ptr-max are marked bold.

### 5.1 Impact of grammar-constrained decoding (RQ1)

This section presents the results of Table 1, i.e., the results w.r.t. 1, discussing basic models with and without grammar-constrained decoding.

The combination of the flan-t5-base base model with grammar-constrained decoding (GCD) yields the overall best results, achieving an *F*_1_ score of 0.413 (±0.13) for the type 2 diabetes and 0.47 (±0.061) for the glaucoma test set. The second best results are achieved by the combination of grammar-constrained decoding and the led-base-16384 base model, albeit performing considerably worse with 0.301 (±0.102) for the type 2 diabetes and 0.292 (±0.12) for the glaucoma test set.

The grammar-constrained decoding approach outperforms the greedy decoding approach on average by far, both for flan-t5-base (0.413 vs. 0.062 for type 2 diabetes and 0.470 vs. 0.045 for glaucoma) and for led-base-16384 (0.301 vs. 0.016 for type 2 diabetes and 0.292 vs. 0.102 for glaucoma).

In spite of achieving the best overall performance, the standard deviation is also highest for the basic + GCD combination in general and for the best-performing model flan-t5-base in particular (0.13 for type 2 diabetes). However, the highest standard deviation for the glaucoma dataset is achieved by the led-base-16384 for basic + GCD with 0.12.

### 5.2 Interplay between grammar-constrained decoding and pointer generators (RQ2)

This section shows the results of Table 2 comparing the pointer models as a whole with the basic baseline using grammar-constrained decoding, i.e., GCD. For this purpose, the best basic scores are shown at the top of Table 2 for both datasets.

In the conducted experiments, the pointer models on average always performed worse than their basic counterparts, with a much higher performance decrease for flan-t5-base (0.413 vs. 0.092 for type 2 diabetes and 0.47 vs. 0.091 for glaucoma) than for led-base-16384 (0.301 vs. 0.263 for type 2 diabetes and 0.292 vs. 0.272 for glaucoma). Thus, in total, pointer models do not outperform vanilla basic models in combination with grammar-constrained decoding. As basicGCD models occupy the first and second place in total, a pointer model can be found in the overall third place. More precisely, this is the ptr-max model using led-base-16384 as a base model with 0.263 (±0.067) for type 2 diabetes and 0.272 (±0.046) for glaucoma.

Examining Table 3, it is also striking that the basic model achieves better mean *F*_1_ scores than both ptr-max and ptr-sum for every single template type. For Table 4, the results are slightly more mixed. For the majority of slots, the performance ranking is the same as for the template, namely basic outperforming ptr-max and ptr-max performing slightly better than ptr-sum. Nevertheless, there are a few exceptions which are marked bold in Table 4. For example, for PValueChangeValue, the pointer model ptr-max achieves a mean *F*_1_ score of 0.08 whereas the basic model only reaches a score of 0.01.

However, there is no slot for which ptr-sum outperforms basic. Additionally, these different performances occur mostly for slots which only have comparably low *F*_1_ scores anyway and is typically paired with a high standard deviation. This may indicate that these exceptions are more due to noise and random fluctuations than to actual architectural differences. In order to further investigate this hypothesis, additional experiments would be necessary, which remain to be done in future work.

### 5.3 Performance of different attention aggregation strategies (RQ3)

This section inspects the results of Table 2 comparing the pointer models with each other in order to determine the best attention aggregation method, i.e., either sum (ptr-sum) or maximum (ptr-max).

In absolute numbers, ptr-max models perform slightly better than ptr-sum models. However, the ptr-sum architecture seems to work a lot better for flan-t5-base than ptr-max (0.092 vs. 0.16 for type 2 diabetes and 0.091 vs. 0.211 for glaucoma) while working reasonably well for led-base-16384, too (0.263 vs. 0.236 for type 2 diabetes and 0.272 vs. 0.216 for glaucoma).

Regarding standard deviation, both pointer model architectures deliver comparable values for type 2 diabetes. For glaucoma, the standard deviation is about twice as large for ptr-sum than for ptr-max, even for both base models (0.046 vs. 0.078 for led-base-16384 and 0.015 vs. 0.084 for flan-t5-base).

Regarding Table 3, ptr-max outperforms ptr-sum for every template on the glaucoma dataset. However, the standard deviation is higher in most cases for the ptr-sum model, indicating that the performance of ptr-sum models is more volatile but can be better than ptr-max in extreme cases. For example, for the Medication template, the mean score with 0.3 vs. 0.27 is comparable for ptr-max and ptr-sum but the standard deviation for ptr-sum is twice as high with 0.06 vs. 0.12, indicating a higher potential to achieve high scores in some cases. Whether these high performance trials can be achieved more consistently with different training parameters is unclear and remains to be investigated in future work.

For Table 4, the results are slightly more mixed for the performance of the different pointer models just as when comparing them to the basic baseline in the previous section. Nevertheless, ptr-max is performing slightly better than ptr-sum usually. However, for slot DeliveryMethod, there is an exception where ptr-sum outperforms ptr-max with a score of 0.11 vs. 0.05.

### 5.4 Ablation study: increasing model size

Although the influence of the model size is not systematically evaluated in this work, we conducted a small ablation study for a single branch of the experiments presented above. Concretely, we trained a basic model of the google/flan-t5-large base model on the glaucoma dataset in the same 30+10 trials fashion described above and evaluated the resulting 10 models both with and without grammar-constrained decoding. With grammar-constrained decoding, i.e., for GCD, the result on the glaucoma test set is an *F*_1_ score of 0.490 (±0.053). With standard greedy decoding, i.e., noGCD, a score of 0.044 (±0.043) is achieved.

Compared to flan-t5-base with 0.47 (±0.061) with GCD and 0.045 (±0.043) with noGCD, this is a slight performance improvement when using grammar-constrained decoding and a very similar or even slightly worse result when using standard greedy decoding. This indicates that increasing model size alone does not solve the problems with reliably generating syntactically correct output sequences. However, more structured evaluations are necessary to test this hypothesis further.

Listing 1 **Case study of an arbitrarily chosen syntax error made by google/flan-t5-large trained on the glaucoma dataset when evaluated without grammar-constrained decoding, i.e., noGCD.**


1   ...
2   [end:hasEndpoint]
3   **[start:hasObservedResult]**
4   After 3 months of treatment...
5   **[end:hasObservedResult]**
6   **[start:hasPValueChangeValue]**
7   After 3 months of treatment...
8   **[end:hasObservedResult]**
9   [start:hasPValueChangeValue]
10   P=0.01
11   [end:hasPValueChangeValue]
12   [end:Outcome]
13   [end:hasOutcome]
14   ...

In Listing 1, an exemplary syntax error made by the fine-tuned google/flan-t5-large during evaluation with the glaucoma test set is shown. Considering the presented output snippet, it is striking that the model generates the first part correctly, i.e., [start:hasObservedResult]After 3 months of treatment...[end:hasObservedResult] is syntactically correct. But after that, some kind of mixture between hasObservedResult and hasPValueChangeValue seems to be generated, as the content is almost identical to the hasObservedResult slot whereas the starting tag, i.e., hasPValueChangeValue, is not. The similar content might be the reason why the model confuses the end tags and chooses hasObservedResult over the correct hasPValueChangeValue. After this, a correct instance of hasPValueChangeValue is then generated with different content and no confusion of the end tag.

Although the type of syntax errors were not systematically evaluated in this work, the presented example appears to be prototypical for the category of errors that is common when looking at unconstrained output. Despite large parts of the generated output being correct and meaningful, there is often just a small error which causes the whole output to end up invalid due to the strict requirements imposed by the context-free grammar. At the same time, this kind of errors can easily be circumvented with grammar-constrained decoding, preserving the validity of the otherwise in large parts useful and correct output. A more structured evaluation of these kinds of mistakes unconstrained models do would be an interesting path for future work.

## 6 Discussion

In this section, we discuss the results presented in the previous section w.r.t. the research questions of this paper and connect our findings to some related work.

Considering the bad performance of almost all noGCD configurations with an absolute performance increase for GCD between 0.425 (glaucoma dataset, flan-t5-base + basic) and 0.091 (glaucoma dataset, flan-t5-base + ptr), this suggests w.r.t. 1 that grammar-constrained decoding helps the models substantially to generate better results and eliminates the burden of having to learn the structure of the data from examples. Thus, grammar-constrained decoding positively affects the performance for the considered structured information extraction task.

This is in line with the results of Geng et al. (2023). However, they have only shown the positive impact of grammar-constrained decoding on pre-trained models. In contrast, we have focused on fine-tuned models and shown that also in this setting grammar-constrained decoding has a very positive impact, in particular in what we have called low-resource settings. Considering the higher amount of data given to the models compared to few-shot prompting, one could have expected the benefit of grammar-constrained decoding to decrease. Instead, our experiments indicate that grammar-constrained decoding can still be useful in fine-tuning settings, at least for the comparably small models that have been tested and the resulting performance increase is similar, if not larger, to the results obtained by Geng et al. (2023). The poor performance without grammar-constrained decoding may also be caused by the usage of special tokens for the start and end tags, which were not part of the pre-training process. Thus, the available training data might have been too small for learning the meaning and structure of these tokens. Whether not using special tokens and instead relying on the existing vocabulary for the structure generation would improve the performance is unclear and remains to be investigated in future work. However, some preliminary experiments indicate that the task is actually easier to learn for the models with special tokens compared to tokenizing the slot start and end tags like regular text.

At the same time, this shows that learning a complex output structure reliably from relatively few examples is still not a trivial task for large language models of the considered size (e.g., ca. 220 million parameters for flan-t5-base and ca. 160 million parameters for led-base-16384). How the size of the used large language model affects this part of the performance remains to be investigated in future work in a more structured way. Our ablation study with flan-t5-large presented in the previous section suggests that the benefit of using grammar-constrained decoding is similarly large even when the model size is increased. However, Geng et al. (2023) use much larger models and appear to get very promising results such that the benefit of grammar-constrained decoding might be smaller for fine-tuning on larger models. These results are also in agreement with Sun et al. (2023), although they primarily explored the validity of SMT solver formulas when varying the model temperature, whereas this work evaluated a domain-specific grammar and varied different architectural properties, yielding a much larger validity difference between constrained and unconstrained decoding. In summary, this illustrates that large language models actually struggle with sticking to strictly-constrained output structures in practice such that having guarantees as provided by grammar-constrained decoding is useful, especially for smaller models or rare output structures but also for larger models.

All in all, this emphasizes that grammar-constrained decoding appears to be beneficial when fine-tuning in low-resource settings for structured information extraction tasks.

Regarding 1, the presented results suggest that pointer generators are not beneficial for structured information extraction tasks in low-resource environments when combined with grammar-constrained decoding. They therefore seem not to be a promising path of future research, at least with the considered models, dataset size, output syntax and overall architecture. In contrast, Lin et al. (2020) successfully generated SQL statements from text using a BERT model in combination with pointer generators, which indicates that pointer generators can be useful in the context of structure generation nevertheless. However, it is not clear which aspect made the pointer generator approach fail in our case, whether it was the dataset size, choice of models, the (compared to SQL) rare output structure or something else. Therefore, a deeper analysis of the errors made by the pointer models and where the differences to the successes of Lin et al. (2020) are is open to be explored in future work.

All in all, this indicates that pointer generator-like behavior seems to hurt performance in structured information extraction tasks in low-resource settings combined with grammar-constrained decoding instead of improving it.

Considering 1, i.e., the two attention aggregation methods, the pointer generator-like behavior with maximum used for attention aggregation ptr-max works better for the (otherwise worse-performing) led-base-16384 base model than for flan-t5-base. However, the pointer generator models with summing as an aggregation function ptr-sum perform comparably well in combination with both base models, although slightly worse in absolute numbers. It is not clear which properties of the architecture cause this difference, both between the pointer models and the basic model as well as between the pointer models ptr-max and ptr-sum, such that this remains to be investigated in future work.

All in all, this means there is no clear winner comparing the maximum and sum function as attention aggregation methods for pointer generator-like models and the choice appears to depend on the used base model as well. Overall, the maximum aggregation function achieved the best scores for both datasets.

## 7 Conclusion

In this work, we have presented a grammar-constrained decoding approach for structured information extraction with fine-tuned generative large language models. Our sequence-to-sequence models predict complex output structures, consisting of nested templates with both textual slots as well as slots again containing templates. The chosen base models google/flan-t5-base and allenai/led-base-16384 have been evaluated in multiple configurations, i.e., with and without support by grammar-constrained decoding as well as with two kinds of supporting pointer generator-like behavior.

We have instantiated the model specifically for PICO element extraction from randomized controlled trials in combination with a domain-specific grammar for that purpose and evaluated all different model configurations w.r.t. two diseases, namely type 2 diabetes and glaucoma.

In summary, our results indicate that grammar-constrained decoding can substantially increase the model performance in low-resource settings for structured information extraction tasks (1) and that pointer generator-like behavior appears not to be beneficial in the considered settings, with varying intensities of performance degradation depending on the model and the chosen attention aggregation function (1). Furthermore, the used attention aggregation method appears to depend on the used model and, in total, the maximum function achieves the best results for both datasets (1).

Evaluating in a structured way how the large language models size affects the performance benefit of grammar-constrained decoding as well as investigating the reasons for the generally bad performance of both pointer generator models ptr-max and ptr-sum are just a few aspects besides many others that still pose interesting open questions which remain to be investigated in future work.

## Data Availability

The datasets presented in this study can be found in online repositories. The names of the repository/repositories and accession number(s) can be found below: https://zenodo.org/doi/10.5281/zenodo.10419785. The code used in this work has been published as (Schmidt and Cimiano, 2024).

## Footnotes

^1^https://huggingface.co/google/flan-t5-base.

^2^https://huggingface.co/allenai/led-base-16384.
