# Supplementary material for: Grammar-constrained decoding for structured information extraction with fine-tuned generative models applied to clinical trial abstracts
Source: Front Artif Intell. 2025 Jan 7;7:1406857. doi: 10.3389/frai.2024.1406857 (PMC11747381; doi:10.3389/frai.2024.1406857)
Supplement: Supplementary file 1 [file Data_Sheet_1.pdf]

Supplementary Material

1 SUPPLEMENTARY DATA

1.1 Lark Grammar

```
hasadverseeffect: "[start:hasAdverseEffect]" outcome "[end:hasAdverseEffect]"
hasfinalnumpatientsarm: "[start:hasFinalNumPatientsArm]" POINT+ "[end:hasFinalNumPatientsArm]"
hasintervention: "[start:hasIntervention]" intervention "[end:hasIntervention]"
hasnumberpatientsarm: "[start:hasNumberPatientsArm]" POINT+ "[end:hasNumberPatientsArm]"
hasoutcome: "[start:hasOutcome]" outcome "[end:hasOutcome]"

arm: "[start:Arm]" _arm* "[end:Arm]"
_arm: hasadverseeffect
| hasfinalnumpatientsarm
| hasintervention
| hasnumberpatientsarm
| hasoutcome

analyseshealthcondition: "[start:analysesHealthCondition]" POINT+ "[end:analysesHealthCondition]"
hasallocationratio: "[start:hasAllocationRatio]" POINT+ "[end:hasAllocationRatio]"
hasarm: "[start:hasArm]" arm "[end:hasArm]"
hasctdesign: "[start:hasCTDesign]" POINT+ "[end:hasCTDesign]"
hasctduration: "[start:hasCTduration]" POINT+ "[end:hasCTduration]"
hasconclusioncomment: "[start:hasConclusionComment]" POINT+ "[end:hasConclusionComment]"
hasdiffbetweengroups: "[start:hasDiffBetweenGroups]" diffbetweengroups "[end:hasDiffBetweenGroups]"
hasfinalnumberpatientsct: "[start:hasFinalNumberPatientsCT]" POINT+ "[end:hasFinalNumberPatientsCT]"
hasnumberpatientsct: "[start:hasNumberPatientsCT]" POINT+ "[end:hasNumberPatientsCT]"
hasobjectivedescription: "[start:hasObjectiveDescription]" POINT+ "[end:hasObjectiveDescription]"
haspopulation: "[start:hasPopulation]" population "[end:hasPopulation]"

clinicaltrial: "[start:ClinicalTrial]" _clinicaltrial* "[end:ClinicalTrial]"
_clinicaltrial: analyseshealthcondition
| hasallocationratio
| hasarm
| hasctdesign
| hasctduration
| hasconclusioncomment
```

```
| hasdiffbetweengroups
| hasfinalnumberpatientsct
| hasnumberpatientsct
| hasobjectivedescription
| haspopulation

hasconfintervaldiff: "[start:hasConfIntervalDiff]" POINT+ "[end:hasConfIntervalDiff]"
hasdiffgroupabsvalue: "[start:hasDiffGroupAbsValue]" POINT+ "[end:hasDiffGroupAbsValue]"
haspvaluediff: "[start:hasPvalueDiff]" POINT+ "[end:hasPvalueDiff]"

diffbetweengroups: "[start:DiffBetweenGroups]" _diffbetweengroups* "[end:DiffBetweenGroups]"
_diffbetweengroups: hasconfintervaldiff
| hasdiffgroupabsvalue
| haspvaluediff

hasaggregationmethod: "[start:hasAggregationMethod]" POINT+ "[end:hasAggregationMethod]"
hasbaselineunit: "[start:hasBaselineUnit]" POINT+ "[end:hasBaselineUnit]"
hasendopointdescription: "[start:hasEndoPointDescription]" POINT+ "[end:hasEndoPointDescription]"
hasmeasurementdevice: "[start:hasMeasurementDevice]" POINT+ "[end:hasMeasurementDevice]"

endpoint: "[start:Endpoint]" _endpoint* "[end:Endpoint]"
_endpoint: hasaggregationmethod
| hasbaselineunit
| hasendopointdescription
| hasmeasurementdevice

hasfrequency: "[start:hasFrequency]" POINT+ "[end:hasFrequency]"
hasmedication: "[start:hasMedication]" medication "[end:hasMedication]"
hasrelativefreqtime: "[start:hasRelativeFreqTime]" POINT+ "[end:hasRelativeFreqTime]"

intervention: "[start:Intervention]" _intervention* "[end:Intervention]"
_intervention: hasfrequency
| hasmedication
| hasrelativefreqtime

hasdeliverymethod: "[start:hasDeliveryMethod]" POINT+ "[end:hasDeliveryMethod]"
hasdosedescription: "[start:hasDoseDescription]" POINT+ "[end:hasDoseDescription]"
hasdoseunit: "[start:hasDoseUnit]" POINT+ "[end:hasDoseUnit]"
hasdosevalue: "[start:hasDoseValue]" POINT+ "[end:hasDoseValue]"
hasdrug: "[start:hasDrug]" POINT+ "[end:hasDrug]"
```

```
medication: ”[start:Medication]” _medication* ”[end:Medication]”
_medication: hasdeliverymethod
| hasdosedescription
| hasdoseunit
| hasdosevalue
| hasdrug

hasbaselinevalue: ”[start:hasBaselineValue]” POINT+ ”[end:hasBaselineValue]”
haschangevalue: ”[start:hasChangeValue]” POINT+ ”[end:hasChangeValue]”
hasconfintervalchangevalue: ”[start:hasConfIntervalChangeValue]” POINT+ ”[end:hasConfIntervalChangeValue]”
hasendpoint: ”[start:hasEndpoint]” endpoint ”[end:hasEndpoint]”
hasnumberaffected: ”[start:hasNumberAffected]” POINT+ ”[end:hasNumberAffected]”
hasobservedresult: ”[start:hasObservedResult]” POINT+ ”[end:hasObservedResult]”
haspvaluechangevalue: ”[start:hasPValueChangeValue]” POINT+ ”[end:hasPValueChangeValue]”
haspercentageaffected: ”[start:hasPercentageAffected]” POINT+ ”[end:hasPercentageAffected]”
hasrelativechangevalue: ”[start:hasRelativeChangeValue]” POINT+ ”[end:hasRelativeChangeValue]”
hasresultmeasuredvalue: ”[start:hasResultMeasuredValue]” POINT+ ”[end:hasResultMeasuredValue]”
hassddevbl: ”[start:hasSdDevBL]” POINT+ ”[end:hasSdDevBL]”
hassddevchangevalue: ”[start:hasSdDevChangeValue]” POINT+ ”[end:hasSdDevChangeValue]”
hassddevresvalue: ”[start:hasSdDevResValue]” POINT+ ”[end:hasSdDevResValue]”
hassubgroupdescription: ”[start:hasSubGroupDescription]” POINT+ ”[end:hasSubGroupDescription]”
hassderrorchangevalue: ”[start:hasSdErrorChangeValue]” POINT+ ”[end:hasSdErrorChangeValue]”
hastimepoint: ”[start:hasTimePoint]” POINT+ ”[end:hasTimePoint]”

outcome: ”[start:Outcome]” _outcome* ”[end:Outcome]”
_outcome: hasbaselinevalue
| haschangevalue
| hasconfintervalchangevalue
| hasendpoint
| hasnumberaffected
| hasobservedresult
| haspvaluechangevalue
| haspercentageaffected
| hasrelativechangevalue
| hasresultmeasuredvalue
| hassddevbl
| hassddevchangevalue
| hassddevresvalue
```

```
| hassubgroupdescription
| hassderrorchangevalue
| hastimepoint

hasavgage: "[start:hasAvgAge]" POINT+ "[end:hasAvgAge]"
hascountry: "[start:hasCountry]" POINT+ "[end:hasCountry]"
hasminage: "[start:hasMinAge]" POINT+ "[end:hasMinAge]"
hasprecondition: "[start:hasPrecondition]" POINT+ "[end:hasPrecondition]"

population: "[start:Population]" _population* "[end:Population]"
_population: hasavgage
| hascountry
| hasminage
| hasprecondition

describes: "[start:describes]" clinicaltrial "[end:describes]"
hasauthor: "[start:hasAuthor]" POINT+ "[end:hasAuthor]"
hasjournal: "[start:hasJournal]" POINT+ "[end:hasJournal]"
haspmid: "[start:hasPMID]" POINT+ "[end:hasPMID]"
haspublicationyear: "[start:hasPublicationYear]" POINT+ "[end:hasPublicationYear]"
hastitle: "[start:hasTitle]" POINT+ "[end:hasTitle]"

publication: "[start:Publication]" describes? _publication* "[end:Publication]"
_publication: hasauthor
| hasjournal
| haspmid
| haspublicationyear
| hastitle
```

Listing 1. Lark EBNF grammar of the data model used for decoding and parsing in our experiments. For decoding, the `POINT` nonterminal is defined using a simple regular expression avoiding matches of `[start:` and `[end:` but allowing arbitrary freetext besides that. For parsing the decoder output, the `POINT` nonterminal is replaced by a definition considering the concrete tokenizer vocabulary instead.

1.2 Additional Evaluation Results

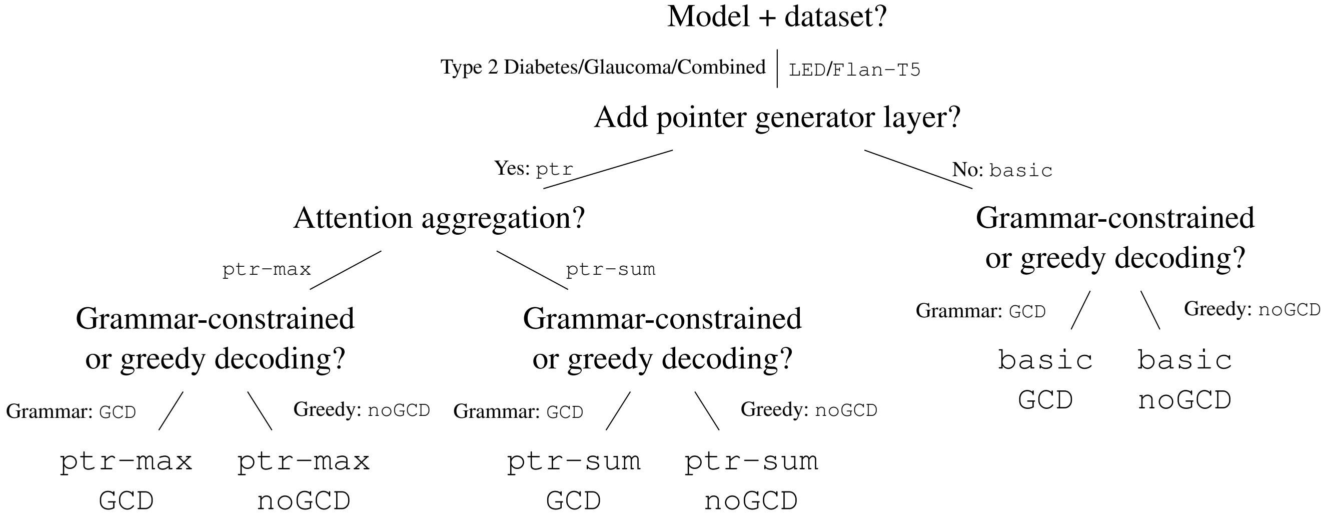

1.2.1 Results for Pointer Generators Including Greedy Decoding

↓Setting Model	Dataset→ Type	Decoding	Type 2 Diabetes Mean F_1 ($\pm\sigma$)	Glaucoma Mean F_1 ($\pm\sigma$)
flan-t5-base	basic	GCD	**0.413 (±0.13)**	**0.47 (±0.061)**
flan-t5-base	basic	noGCD	0.062 (±0.041)	0.045 (±0.043)
flan-t5-base	ptr-max	GCD	0.092 (±0.075)	0.091 (±0.015)
flan-t5-base	ptr-max	noGCD	0.0 (±0.0)	0.0 (±0.0)
flan-t5-base	ptr-sum	GCD	0.16 (±0.074)	0.211(±0.084)
flan-t5-base	ptr-sum	noGCD	0.0 (±0.0)	0.0 (±0.0)
led-base-16384	basic	GCD	0.301 (±0.102)	0.292 (±0.12)
led-base-16384	basic	noGCD	0.016 (±0.029)	0.102 (±0.049)
led-base-16384	ptr-max	GCD	0.263 (±0.067)	0.272 (±0.046)
led-base-16384	ptr-max	noGCD	0.009 (±0.018)	0.041 (±0.059)
led-base-16384	ptr-sum	GCD	0.236 (±0.064)	0.216 (±0.078)
led-base-16384	ptr-sum	noGCD	0.0 (±0.0)	0.0(±0.0)

Table S1. Mean and standard deviation σ of test F_1 scores across 10 models trained on *either the type 2 diabetes or glaucoma dataset* using best-performing (F_1 on validation dataset) configuration found in 30 trials of hyperparameter optimization. Numbers rounded to three decimal places, best configuration of each disease marked bold.

1.2.2 Generated Instance Counts Per Template for Best Model vs. Ground Truth Type 2 Diabetes basic GCD

Template Name	Mean GT Count	Mean Predicted Count	Abs Diff
Arm	2.00	1.86 (±0.19)	0.14 (±0.19)
ClinicalTrial	1.00	1.0 (±0.0)	0.0 (±0.0)
DiffBetweenGroups	2.89	2.49 (±0.93)	0.6 (±0.86)
Endpoint	5.85	9.67 (±1.24)	3.82 (±1.24)
EvidenceQuality	1.00	0.0 (±0.0)	1.0 (±0.0)
Intervention	2.10	1.65 (±0.19)	0.46 (±0.19)
Medication	2.20	1.64 (±0.18)	0.57 (±0.18)
Outcome	10.35	9.69 (±1.25)	1.16 (±0.75)
Population	0.95	0.74 (±0.29)	0.22 (±0.3)
Publication	1.00	1.0 (±0.0)	0.0 (±0.0)

1.2.3 Generated Instance Counts Per Template for Best Model vs. Ground Truth Type 2 Diabetes basic noGCD

Template Name	Mean GT Count	Mean Predicted Count	Abs Diff
Arm	2.00	0.12 (±0.08)	1.88 (±0.08)
ClinicalTrial	1.00	0.06 (±0.04)	0.94 (±0.04)
DiffBetweenGroups	3.06	0.23 (±0.16)	2.83 (±0.16)
Endpoint	5.85	0.45 (±0.32)	5.4 (±0.32)
EvidenceQuality	1.00	0.0 (±0.0)	1.0 (±0.0)
Intervention	2.10	0.11 (±0.07)	2.0 (±0.07)
Medication	2.20	0.11 (±0.07)	2.1 (±0.07)
Outcome	10.35	0.45 (±0.32)	9.9 (±0.32)
Population	1.00	0.06 (±0.04)	0.94 (±0.04)
Publication	1.00	0.06 (±0.04)	0.94 (±0.04)

1.2.4 Generated Instance Counts Per Template for Best Model vs. Ground Truth Type 2 Diabetes ptr-max GCD

Template Name	Mean GT Count	Mean Predicted Count	Abs Diff
Arm	2.00	1.66 (±0.28)	0.37 (±0.25)
ClinicalTrial	1.00	1.0 (±0.0)	0.0 (±0.0)
DiffBetweenGroups	2.89	2.14 (±0.81)	1.03 (±0.43)
Endpoint	5.85	7.58 (±2.88)	2.7 (±1.9)
EvidenceQuality	1.00	0.0 (±0.0)	1.0 (±0.0)
Intervention	2.10	1.36 (±0.34)	0.74 (±0.34)
Medication	2.20	1.34 (±0.34)	0.86 (±0.34)
Outcome	10.35	7.62 (±2.89)	3.15 (±2.35)
Population	0.95	0.42 (±0.2)	0.55 (±0.2)
Publication	1.00	1.0 (±0.0)	0.0 (±0.0)

1.2.5 Generated Instance Counts Per Template for Best Model vs. Ground Truth Type 2 Diabetes ptr-max noGCD

Template Name	Mean GT Count	Mean Predicted Count	Abs Diff
Arm	2.00	0.03 (±0.06)	1.97 (±0.06)
ClinicalTrial	1.00	0.01 (±0.02)	0.99 (±0.02)
DiffBetweenGroups	3.06	0.02 (±0.05)	3.03 (±0.05)
Endpoint	5.85	0.09 (±0.19)	5.76 (±0.19)
EvidenceQuality	1.00	0.0 (±0.0)	1.0 (±0.0)
Intervention	2.10	0.03 (±0.05)	2.08 (±0.05)
Medication	2.20	0.03 (±0.05)	2.18 (±0.05)
Outcome	10.35	0.09 (±0.19)	10.26 (±0.19)
Population	1.00	0.01 (±0.02)	0.99 (±0.02)
Publication	1.00	0.01 (±0.02)	0.99 (±0.02)

1.2.6 Generated Instance Counts Per Template for Best Model vs. Ground Truth Glaucoma basic GCD

Template Name	Mean GT Count	Mean Predicted Count	Abs Diff
Arm	2.00	1.91 (±0.13)	0.1 (±0.11)
ClinicalTrial	1.00	1.0 (±0.0)	0.0 (±0.0)
DiffBetweenGroups	1.70	1.65 (±0.2)	0.19 (±0.07)
Endpoint	2.48	5.71 (±1.5)	3.24 (±1.5)
EvidenceQuality	1.00	0.0 (±0.0)	1.0 (±0.0)
Intervention	2.19	1.81 (±0.21)	0.38 (±0.21)
Medication	2.33	1.84 (±0.22)	0.5 (±0.22)
Outcome	5.05	5.79 (±1.5)	1.08 (±1.25)
Population	0.95	0.8 (±0.15)	0.16 (±0.14)
Publication	1.00	1.0 (±0.0)	0.0 (±0.0)

1.2.7 Generated Instance Counts Per Template for Best Model vs. Ground Truth Glaucoma basic noGCD

Template Name	Mean GT Count	Mean Predicted Count	Abs Diff
Arm	2.00	0.22 (±0.12)	1.78 (±0.12)
ClinicalTrial	1.00	0.11 (±0.06)	0.89 (±0.06)
DiffBetweenGroups	1.89	0.29 (±0.23)	1.56 (±0.23)
Endpoint	2.48	0.46 (±0.28)	2.02 (±0.28)
EvidenceQuality	1.00	0.0 (±0.0)	1.0 (±0.0)
Intervention	2.19	0.22 (±0.11)	1.97 (±0.11)
Medication	2.33	0.23 (±0.12)	2.1 (±0.12)
Outcome	5.05	0.46 (±0.28)	4.59 (±0.28)
Population	1.00	0.12 (±0.06)	0.88 (±0.06)
Publication	1.00	0.11 (±0.06)	0.89 (±0.06)

1.2.8 Generated Instance Counts Per Template for Best Model vs. Ground Truth Glaucoma ptr-max GCD

Template Name	Mean GT Count	Mean Predicted Count	Abs Diff
Arm	2.00	1.5 (±0.2)	0.5 (±0.2)
ClinicalTrial	1.00	1.0 (±0.0)	0.0 (±0.0)
DiffBetweenGroups	1.79	1.51 (±0.96)	0.86 (±0.52)
Endpoint	2.48	5.43 (±1.33)	2.96 (±1.33)
EvidenceQuality	1.00	0.0 (±0.0)	1.0 (±0.0)
Intervention	2.19	1.46 (±0.21)	0.73 (±0.21)
Medication	2.33	1.43 (±0.21)	0.9 (±0.21)
Outcome	5.05	5.45 (±1.32)	1.08 (±0.8)
Population	1.00	0.36 (±0.14)	0.63 (±0.15)
Publication	1.00	1.0 (±0.0)	0.0 (±0.0)

1.2.9 Generated Instance Counts Per Template for Best Model vs. Ground Truth Glaucoma ptr-max noGCD

Template Name	Mean GT Count	Mean Predicted Count	Abs Diff
Arm	2.00	0.09 (±0.13)	1.91 (±0.13)
ClinicalTrial	1.00	0.04 (±0.06)	0.96 (±0.06)
DiffBetweenGroups	1.89	0.11 (±0.17)	1.77 (±0.19)
Endpoint	2.48	0.19 (±0.29)	2.29 (±0.29)
EvidenceQuality	1.00	0.0 (±0.0)	1.0 (±0.0)
Intervention	2.19	0.09 (±0.13)	2.1 (±0.13)
Medication	2.33	0.09 (±0.13)	2.24 (±0.13)
Outcome	5.05	0.19 (±0.29)	4.86 (±0.29)
Population	1.00	0.04 (±0.06)	0.95 (±0.07)
Publication	1.00	0.04 (±0.06)	0.96 (±0.06)

1.2.10 Generated Instance Counts Per Template for Best Model vs. Ground Truth Type 2 Diabetes ptr-sum GCD

Template Name	Mean GT Count	Mean Predicted Count	Abs Diff
Arm	2.00	1.42 (±0.18)	0.58 (±0.18)
ClinicalTrial	1.00	1.0 (±0.0)	0.0 (±0.0)
DiffBetweenGroups	3.06	1.78 (±1.42)	1.73 (±0.68)
Endpoint	5.85	5.79 (±1.57)	1.26 (±0.83)
EvidenceQuality	1.00	0.0 (±0.0)	1.0 (±0.0)
Intervention	2.10	1.1 (±0.26)	1.0 (±0.26)
Medication	2.20	1.04 (±0.29)	1.16 (±0.29)
Outcome	10.35	5.85 (±1.55)	4.5 (±1.55)
Population	1.00	0.28 (±0.17)	0.71 (±0.18)
Publication	1.00	1.0 (±0.0)	0.0 (±0.0)

1.2.11 Generated Instance Counts Per Template for Best Model vs. Ground Truth Type 2 Diabetes ptr-sum noGCD

Template Name	Mean GT Count	Mean Predicted Count	Abs Diff
Arm	2.00	0 (±0.0)	2.0 (±0.0)
ClinicalTrial	1.00	0 (±0.0)	1.0 (±0.0)
DiffBetweenGroups	3.06	0 (±0.0)	3.06 (±0.0)
Endpoint	5.85	0 (±0.0)	5.85 (±0.0)
EvidenceQuality	1.00	0 (±0.0)	1.0 (±0.0)
Intervention	2.10	0 (±0.0)	2.1 (±0.0)
Medication	2.20	0 (±0.0)	2.2 (±0.0)
Outcome	10.35	0 (±0.0)	10.35 (±0.0)
Population	1.00	0 (±0.0)	1.0 (±0.0)
Publication	1.00	0 (±0.0)	1.0 (±0.0)

1.2.12 Generated Instance Counts Per Template for Best Model vs. Ground Truth Glaucoma ptr-sum GCD

Template Name	Mean GT Count	Mean Predicted Count	Abs Diff
Arm	2.00	1.21 (±0.4)	0.79 (±0.4)
ClinicalTrial	1.00	1.0 (±0.0)	0.0 (±0.0)
DiffBetweenGroups	1.89	0.78 (±0.71)	1.2 (±0.45)
Endpoint	2.48	3.04 (±1.99)	1.19 (±1.66)
EvidenceQuality	1.00	0.0 (±0.0)	1.0 (±0.0)
Intervention	2.19	0.94 (±0.38)	1.25 (±0.38)
Medication	2.33	0.89 (±0.37)	1.44 (±0.37)
Outcome	5.05	3.1 (±1.98)	2.54 (±0.99)
Population	1.00	0.2 (±0.13)	0.79 (±0.14)
Publication	1.00	1.0 (±0.0)	0.0 (±0.0)

1.2.13 Generated Instance Counts Per Template for Best Model vs. Ground Truth Glaucoma ptr-sum noGCD

Template Name	Mean GT Count	Mean Predicted Count	Abs Diff
Arm	2.00	0 (±0.0)	2.0 (±0.0)
ClinicalTrial	1.00	0 (±0.0)	1.0 (±0.0)
DiffBetweenGroups	1.89	0 (±0.0)	1.89 (±0.0)
Endpoint	2.48	0 (±0.0)	2.48 (±0.0)
EvidenceQuality	1.00	0 (±0.0)	1.0 (±0.0)
Intervention	2.19	0 (±0.0)	2.19 (±0.0)
Medication	2.33	0 (±0.0)	2.33 (±0.0)
Outcome	5.05	0 (±0.0)	5.05 (±0.0)
Population	1.00	0 (±0.0)	1.0 (±0.0)
Publication	1.00	0 (±0.0)	1.0 (±0.0)

1.2.14 Scores Per Template for Best Models

Template Name	Type 2 Diabetes F_1					
	basic GCD	basic noGCD	ptr-max GCD	ptr-max noGCD	ptr-sum GCD	ptr-sum noGCD
Arm	0.42 (±0.23)	0.03 (±0.06)	0.21 (±0.11)	0.01 (±0.02)	0.19 (±0.08)	0.0 (±0.0)
ClinicalTrial	0.56 (±0.16)	0.09 (±0.06)	0.33 (±0.04)	0.01 (±0.02)	0.31 (±0.09)	0.0 (±0.0)
DiffBetweenGroups	0.27 (±0.11)	0.03 (±0.03)	0.12 (±0.06)	0.0 (±0.01)	0.14 (±0.06)	0.0 (±0.0)
Endpoint	0.36 (±0.11)	0.06 (±0.05)	0.23 (±0.06)	0.01 (±0.03)	0.23 (±0.07)	0.0 (±0.0)
Intervention	0.51 (±0.14)	0.08 (±0.05)	0.31 (±0.06)	0.01 (±0.02)	0.35 (±0.06)	0.0 (±0.0)
Medication	0.38 (±0.08)	0.06 (±0.04)	0.27 (±0.06)	0.01 (±0.02)	0.25 (±0.04)	0.0 (±0.0)
Outcome	0.12 (±0.05)	0.01 (±0.01)	0.06 (±0.02)	0.0 (±0.0)	0.06 (±0.02)	0.0 (±0.0)
Population	0.29 (±0.13)	0.03 (±0.02)	0.12 (±0.08)	0.0 (±0.01)	0.09 (±0.06)	0.0 (±0.0)
Publication	0.66 (±0.25)	0.1 (±0.07)	0.37 (±0.14)	0.02 (±0.04)	0.0 (±0.0)	0.23 (±0.14)

Template Name	Glaucoma F_1					
	basic GCD	basic noGCD	ptr-max GCD	ptr-max noGCD	ptr-sum GCD	ptr-sum noGCD
Arm	0.21 (±0.08)	0.02 (±0.03)	0.07 (±0.06)	0.0 (±0.0)	0.05 (±0.07)	0.0 (±0.0)
ClinicalTrial	0.53 (±0.05)	0.11 (±0.06)	0.3 (±0.07)	0.04 (±0.07)	0.22 (±0.09)	0.0 (±0.0)
DiffBetweenGroups	0.15 (±0.08)	0.05 (±0.05)	0.07 (±0.04)	0.02 (±0.05)	0.04 (±0.03)	0.0 (±0.0)
Endpoint	0.33 (±0.06)	0.09 (±0.04)	0.22 (±0.03)	0.04 (±0.05)	0.19 (±0.07)	0.0 (±0.0)
Intervention	0.49 (±0.1)	0.04 (±0.03)	0.23 (±0.07)	0.02 (±0.04)	0.21 (±0.1)	0.0 (±0.0)
Medication	0.51 (±0.11)	0.05 (±0.07)	0.3 (±0.06)	0.04 (±0.05)	0.27 (±0.12)	0.0 (±0.0)
Outcome	0.26 (±0.05)	0.04 (±0.03)	0.1 (±0.04)	0.01 (±0.02)	0.09 (±0.05)	0.0 (±0.0)
Population	0.47 (±0.06)	0.08 (±0.05)	0.25 (±0.08)	0.04 (±0.05)	0.16 (±0.1)	0.0 (±0.0)
Publication	0.69 (±0.06)	0.17 (±0.08)	0.34 (±0.1)	0.07 (±0.1)	0.27 (±0.16)	0.0 (±0.0)

1.2.15 Generated Instance Counts Per Template for Best Models

	Type 2 Diabetes MAD					
Template Name	basic GCD	basic noGCD	ptr-max GCD	ptr-max noGCD	ptr-sum GCD	ptr-sum noGCD
Arm	0.14 (±0.19)	1.88 (±0.08)	0.37 (±0.25)	1.97 (±0.06)	0.58 (±0.18)	2.0 (±0.0)
ClinicalTrial	0.0 (±0.0)	0.94 (±0.04)	0.0 (±0.0)	0.99 (±0.02)	0.0 (±0.0)	1.0 (±0.0)
DiffBetweenGroups	0.6 (±0.86)	2.83 (±0.16)	1.03 (±0.43)	3.03 (±0.05)	1.73 (±0.68)	3.06 (±0.0)
Endpoint	3.82 (±1.24)	5.4 (±0.32)	2.7 (±1.9)	5.76 (±0.19)	1.26 (±0.83)	5.85 (±0.0)
EvidenceQuality	1.0 (±0.0)	1.0 (±0.0)	1.0 (±0.0)	1.0 (±0.0)	1.0 (±0.0)	1.0 (±0.0)
Intervention	0.46 (±0.19)	2.0 (±0.07)	0.74 (±0.34)	2.08 (±0.05)	1.0 (±0.26)	2.1 (±0.0)
Medication	0.57 (±0.18)	2.1 (±0.07)	0.86 (±0.34)	2.18 (±0.05)	1.16 (±0.29)	2.2 (±0.0)
Outcome	1.16 (±0.75)	9.9 (±0.32)	3.15 (±2.35)	10.26 (±0.19)	4.5 (±1.55)	10.35 (±0.0)
Population	0.22 (±0.3)	0.94 (±0.04)	0.55 (±0.2)	0.99 (±0.02)	0.71 (±0.18)	1.0 (±0.0)
Publication	0.0 (±0.0)	0.94 (±0.04)	0.0 (±0.0)	0.99 (±0.02)	0.0 (±0.0)	1.0 (±0.0)

	Glaucoma MAD					
Template Name	basic GCD	basic noGCD	ptr-max GCD	ptr-max noGCD	ptr-sum GCD	ptr-sum noGCD
Arm	0.1 (±0.11)	1.78 (±0.12)	0.5 (±0.2)	1.91 (±0.13)	0.79 (±0.4)	2.0 (±0.0)
ClinicalTrial	0.0 (±0.0)	0.89 (±0.06)	0.0 (±0.0)	0.96 (±0.06)	0.0 (±0.0)	1.0 (±0.0)
DiffBetweenGroups	0.19 (±0.07)	1.56 (±0.23)	0.86 (±0.52)	1.77 (±0.19)	1.2 (±0.45)	1.89 (±0.0)
Endpoint	3.24 (±1.5)	2.02 (±0.28)	2.96 (±1.33)	2.29 (±0.29)	1.19 (±1.66)	2.48 (±0.0)
EvidenceQuality	1.0 (±0.0)	1.0 (±0.0)	1.0 (±0.0)	1.0 (±0.0)	1.0 (±0.0)	1.0 (±0.0)
Intervention	0.38 (±0.21)	1.97 (±0.11)	0.73 (±0.21)	2.1 (±0.13)	1.25 (±0.38)	2.19 (±0.0)
Medication	0.5 (±0.22)	2.1 (±0.12)	0.9 (±0.21)	2.24 (±0.13)	1.44 (±0.37)	2.33 (±0.0)
Outcome	1.08 (±1.25)	4.59 (±0.28)	1.08 (±0.8)	4.86 (±0.29)	2.54 (±0.99)	5.05 (±0.0)
Population	0.16 (±0.14)	0.88 (±0.06)	0.63 (±0.15)	0.95 (±0.07)	0.79 (±0.14)	1.0 (±0.0)
Publication	0.0 (±0.0)	0.89 (±0.06)	0.0 (±0.0)	0.96 (±0.06)	0.0 (±0.0)	1.0 (±0.0)

1.2.16 Scores Per Slot for Best Models

Slot Name	Type 2 Diabetes F_1					
	basic GCD	basic noGCD	ptr-max GCD	ptr-max noGCD	ptr-sum GCD	ptr-sum noGCD
AggregationMethod	0.39 (±0.16)	0.07 (±0.09)	0.2 (±0.1)	0.02 (±0.05)	0.2 (±0.09)	0.0 (±0.0)
AllocationRatio	0.58 (±0.29)	0.11 (±0.15)	0.28 (±0.18)	0.0 (±0.0)	0.51 (±0.17)	0.0 (±0.0)
Author	0.67 (±0.24)	0.11 (±0.08)	0.41 (±0.16)	0.02 (±0.05)	0.29 (±0.18)	0.0 (±0.0)
AvgAge	0.1 (±0.21)	0.0 (±0.0)	0.04 (±0.14)	0.0 (±0.0)	0.0 (±0.0)	0.0 (±0.0)
BaselineUnit	0.38 (±0.09)	0.06 (±0.04)	0.26 (±0.1)	0.01 (±0.02)	0.26 (±0.09)	0.0 (±0.0)
BaselineValue	0.32 (±0.14)	0.02 (±0.04)	0.14 (±0.11)	0.0 (±0.0)	0.14 (±0.08)	0.0 (±0.0)
CTDesign	0.59 (±0.16)	0.07 (±0.07)	0.41 (±0.1)	0.0 (±0.01)	0.34 (±0.13)	0.0 (±0.0)
CTduration	0.73 (±0.2)	0.12 (±0.07)	0.42 (±0.12)	0.02 (±0.04)	0.32 (±0.11)	0.0 (±0.0)
ChangeValue	0.21 (±0.1)	0.04 (±0.04)	0.17 (±0.09)	0.0 (±0.01)	0.16 (±0.07)	0.0 (±0.0)
ConclusionComment	0.5 (±0.19)	0.07 (±0.06)	0.2 (±0.08)	0.01 (±0.02)	0.15 (±0.09)	0.0 (±0.0)
ConfIntervalChangeValue	0.0 (±0.0)	0.0 (±0.0)	0.07 (±0.21)	0.0 (±0.0)	0.0 (±0.0)	0.0 (±0.0)
ConfIntervalDiff	0.27 (±0.12)	0.02 (±0.05)	0.06 (±0.08)	0.0 (±0.0)	0.12 (±0.09)	0.0 (±0.0)
Country	0.5 (±0.22)	0.07 (±0.05)	0.27 (±0.12)	0.0 (±0.0)	0.23 (±0.14)	0.0 (±0.0)
DeliveryMethod	0.0 (±0.0)	0.0 (±0.0)	0.0 (±0.0)	0.0 (±0.0)	0.0 (±0.0)	0.0 (±0.0)
DiffGroupAbsValue	0.3 (±0.14)	0.04 (±0.06)	0.06 (±0.04)	0.01 (±0.02)	0.08 (±0.08)	0.0 (±0.0)
DoseDescription	0.02 (±0.05)	0.0 (±0.0)	0.0 (±0.0)	0.0 (±0.0)	0.0 (±0.0)	0.0 (±0.0)
DoseUnit	0.67 (±0.11)	0.1 (±0.07)	0.55 (±0.11)	0.01 (±0.03)	0.44 (±0.05)	0.0 (±0.0)
DoseValue	0.65 (±0.17)	0.11 (±0.07)	0.39 (±0.14)	0.01 (±0.03)	0.39 (±0.09)	0.0 (±0.0)
Drug	0.59 (±0.15)	0.07 (±0.06)	0.44 (±0.12)	0.01 (±0.03)	0.41 (±0.08)	0.0 (±0.0)
Duration	-	-	-	-	-	-
EndoPointDescription	0.32 (±0.09)	0.06 (±0.04)	0.23 (±0.04)	0.01 (±0.02)	0.24 (±0.03)	0.0 (±0.0)
FinalNumPatientsArm	0.0 (±0.0)	-	0.0 (±0.0)	-	0.0 (±0.0)	-
FinalNumberPatientsCT	-	-	-	-	-	-
Frequency	0.51 (±0.14)	0.08 (±0.05)	0.31 (±0.06)	0.01 (±0.02)	0.35 (±0.06)	0.0 (±0.0)
Journal	0.69 (±0.26)	0.1 (±0.07)	0.39 (±0.15)	0.02 (±0.04)	0.31 (±0.18)	0.0 (±0.0)
MeasurementDevice	-	-	-	-	-	-
MinAge	0.34 (±0.2)	0.0 (±0.0)	0.03 (±0.11)	0.0 (±0.0)	0.0 (±0.0)	0.0 (±0.0)
NumberAffected	0.0 (±0.0)	0.0 (±0.0)	0.03 (±0.07)	0.0 (±0.0)	0.0 (±0.0)	0.0 (±0.0)
NumberPatientsArm	0.51 (±0.24)	0.03 (±0.06)	0.3 (±0.11)	0.01 (±0.02)	0.28 (±0.14)	0.0 (±0.0)

Continued on next page

Slot Name	Type 2 Diabetes F_1 basic GCD	basic noGCD	ptr-max GCD	ptr-max noGCD	ptr-sum GCD	ptr-sum noGCD
NumberPatientsCT	0.46 (±0.17)	0.11 (±0.07)	0.27 (±0.12)	0.01 (±0.04)	0.18 (±0.16)	0.0 (±0.0)
ObjectiveDescription	0.32 (±0.12)	0.05 (±0.05)	0.2 (±0.07)	0.01 (±0.02)	0.11 (±0.09)	0.0 (±0.0)
ObservedResult	0.03 (±0.02)	0.0 (±0.0)	0.02 (±0.03)	0.0 (±0.0)	0.03 (±0.03)	0.0 (±0.0)
PMID	0.64 (±0.24)	0.11 (±0.07)	0.24 (±0.1)	0.02 (±0.04)	0.16 (±0.11)	0.0 (±0.0)
PValueChangeValue	0.12 (±0.11)	0.0 (±0.0)	0.02 (±0.05)	0.0 (±0.0)	0.06 (±0.07)	0.0 (±0.0)
PercentageAffected	0.4 (±0.18)	0.07 (±0.08)	0.22 (±0.06)	0.0 (±0.01)	0.24 (±0.08)	0.0 (±0.0)
Precondition	0.23 (±0.11)	0.05 (±0.04)	0.14 (±0.11)	0.01 (±0.02)	0.13 (±0.1)	0.0 (±0.0)
PublicationYear	0.78 (±0.29)	0.11 (±0.07)	0.46 (±0.19)	0.02 (±0.04)	0.32 (±0.19)	0.0 (±0.0)
PvalueDiff	0.25 (±0.1)	0.03 (±0.04)	0.23 (±0.1)	0.01 (±0.02)	0.23 (±0.09)	0.0 (±0.0)
RelativeChangeValue	0.0 (±0.0)	0.0 (±0.0)	0.0 (±0.0)	0.0 (±0.0)	0.0 (±0.0)	0.0 (±0.0)
RelativeFreqTime	-	-	-	-	-	-
ResultMeasuredValue	0.16 (±0.1)	0.0 (±0.0)	0.04 (±0.05)	0.0 (±0.0)	0.1 (±0.07)	0.0 (±0.0)
SdDevBL	0.09 (±0.12)	0.0 (±0.0)	0.02 (±0.05)	0.0 (±0.0)	0.0 (±0.0)	0.0 (±0.0)
SdDevChangeValue	0.0 (±0.0)	0.0 (±0.0)	0.0 (±0.0)	0.0 (±0.0)	0.0 (±0.0)	0.0 (±0.0)
SdDevResValue	0.12 (±0.12)	0.0 (±0.0)	0.0 (±0.0)	0.0 (±0.0)	0.05 (±0.08)	0.0 (±0.0)
SdErrorChangeValue	-	-	-	-	-	-
SubGroupDescription	0.0 (±0.0)	0.0 (±0.0)	0.0 (±0.0)	0.0 (±0.0)	0.01 (±0.04)	0.0 (±0.0)
TimePoint	0.19 (±0.09)	0.01 (±0.02)	0.09 (±0.07)	0.0 (±0.0)	0.04 (±0.06)	0.0 (±0.0)
Title	0.52 (±0.22)	0.09 (±0.07)	0.37 (±0.14)	0.02 (±0.04)	0.27 (±0.15)	0.0 (±0.0)
analysesHealthCondition	0.78 (±0.09)	0.09 (±0.07)	0.55 (±0.04)	0.01 (±0.03)	0.55 (±0.04)	0.0 (±0.0)
Total Micro F_1 Score	0.41 (±0.13)	0.06 (±0.04)	0.26 (±0.07)	0.01 (±0.02)	0.24 (±0.06)	0.0 (±0.0)

Slot Name	Glaucoma F_1 basic GCD	basic noGCD	ptr-max GCD	ptr-max noGCD	ptr-sum GCD	ptr-sum noGCD
AggregationMethod	0.52 (±0.08)	0.14 (±0.08)	0.34 (±0.07)	0.06 (±0.08)	0.31 (±0.13)	0.0 (±0.0)
AllocationRatio	-	-	-	-	-	-
Author	0.61 (±0.04)	0.16 (±0.08)	0.34 (±0.11)	0.06 (±0.09)	0.21 (±0.12)	0.0 (±0.0)

Continued on next page

Slot Name	Glaucoma F_1					
	basic GCD	basic noGCD	ptr-max GCD	ptr-max noGCD	ptr-sum GCD	ptr-sum noGCD
AvgAge	-	-	-	-	-	-
BaselineUnit	0.55 (±0.06)	0.16 (±0.08)	0.38 (±0.04)	0.07 (±0.1)	0.3 (±0.1)	0.0 (±0.0)
BaselineValue	0.47 (±0.17)	0.07 (±0.1)	0.18 (±0.1)	0.02 (±0.05)	0.19 (±0.16)	0.0 (±0.0)
CTDesign	0.63 (±0.05)	0.16 (±0.08)	0.39 (±0.09)	0.06 (±0.09)	0.21 (±0.13)	0.0 (±0.0)
CTduration	0.68 (±0.09)	0.12 (±0.08)	0.32 (±0.1)	0.06 (±0.1)	0.25 (±0.14)	0.0 (±0.0)
ChangeValue	0.43 (±0.07)	0.07 (±0.07)	0.21 (±0.05)	0.02 (±0.05)	0.21 (±0.09)	0.0 (±0.0)
ConclusionComment	0.59 (±0.06)	0.1 (±0.05)	0.25 (±0.1)	0.04 (±0.06)	0.14 (±0.09)	0.0 (±0.0)
ConfIntervalChangeValue	-	-	-	-	-	-
ConfIntervalDiff	0.11 (±0.14)	0.04 (±0.08)	0.02 (±0.06)	0.02 (±0.06)	0.0 (±0.0)	0.0 (±0.0)
Country	0.76 (±0.1)	0.14 (±0.09)	0.39 (±0.12)	0.06 (±0.09)	0.25 (±0.14)	0.0 (±0.0)
DeliveryMethod	0.23 (±0.2)	0.0 (±0.0)	0.05 (±0.11)	0.0 (±0.0)	0.11 (±0.14)	0.0 (±0.0)
DiffGroupAbsValue	0.11 (±0.13)	0.03 (±0.07)	0.04 (±0.09)	0.02 (±0.05)	0.02 (±0.06)	0.0 (±0.0)
DoseDescription	-	-	-	-	-	-
DoseUnit	0.69 (±0.13)	0.09 (±0.14)	0.51 (±0.11)	0.05 (±0.08)	0.45 (±0.2)	0.0 (±0.0)
DoseValue	0.65 (±0.12)	0.05 (±0.1)	0.36 (±0.05)	0.04 (±0.07)	0.31 (±0.14)	0.0 (±0.0)
Drug	0.45 (±0.07)	0.07 (±0.06)	0.29 (±0.07)	0.05 (±0.07)	0.23 (±0.1)	0.0 (±0.0)
Duration	-	0.0 (±0.0)	-	-	-	0.0 (±0.0)
EndoPointDescription	0.21 (±0.06)	0.05 (±0.03)	0.16 (±0.04)	0.01 (±0.03)	0.16 (±0.06)	0.0 (±0.0)
FinalNumPatientsArm	0.03 (±0.11)	0.0 (±0.0)	0.0 (±0.0)	0.0 (±0.0)	0.0 (±0.0)	0.0 (±0.0)
FinalNumberPatientsCT	0.11 (±0.16)	0.0 (±0.0)	0.05 (±0.11)	0.0 (±0.0)	0.0 (±0.0)	0.0 (±0.0)
Frequency	0.67 (±0.06)	0.11 (±0.08)	0.4 (±0.08)	0.05 (±0.08)	0.37 (±0.12)	0.0 (±0.0)
Journal	0.66 (±0.07)	0.18 (±0.09)	0.36 (±0.1)	0.07 (±0.1)	0.24 (±0.16)	0.0 (±0.0)
MeasurementDevice	0.06 (±0.13)	0.0 (±0.0)	0.0 (±0.0)	0.0 (±0.0)	0.0 (±0.0)	0.0 (±0.0)
MinAge	-	-	-	-	-	-
NumberAffected	0.37 (±0.27)	0.0 (±0.0)	0.01 (±0.02)	0.0 (±0.0)	0.03 (±0.06)	0.0 (±0.0)
NumberPatientsArm	0.38 (±0.2)	0.03 (±0.07)	0.13 (±0.13)	0.0 (±0.0)	0.1 (±0.15)	0.0 (±0.0)
NumberPatientsCT	0.48 (±0.07)	0.12 (±0.11)	0.27 (±0.19)	0.05 (±0.08)	0.23 (±0.16)	0.0 (±0.0)
ObjectiveDescription	0.36 (±0.09)	0.13 (±0.08)	0.24 (±0.06)	0.04 (±0.06)	0.12 (±0.1)	0.0 (±0.0)
ObservedResult	0.01 (±0.02)	0.0 (±0.0)	0.02 (±0.02)	0.0 (±0.0)	0.01 (±0.02)	0.0 (±0.0)

Continued on next page

Slot Name	Glaucoma F_1					
	basic GCD	basic noGCD	ptr-max GCD	ptr-max noGCD	ptr-sum GCD	ptr-sum noGCD
PMID	0.76 (±0.07)	0.15 (±0.09)	0.32 (±0.12)	0.07 (±0.1)	0.21 (±0.14)	0.0 (±0.0)
PValueChangeValue	0.01 (±0.04)	0.0 (±0.0)	0.08 (±0.13)	0.02 (±0.06)	0.03 (±0.07)	0.0 (±0.0)
PercentageAffected	0.19 (±0.1)	0.02 (±0.04)	0.06 (±0.05)	0.02 (±0.06)	0.05 (±0.07)	0.0 (±0.0)
Precondition	0.18 (±0.06)	0.02 (±0.04)	0.12 (±0.06)	0.01 (±0.03)	0.06 (±0.07)	0.0 (±0.0)
PublicationYear	0.88 (±0.09)	0.19 (±0.09)	0.36 (±0.12)	0.08 (±0.11)	0.29 (±0.18)	0.0 (±0.0)
PvalueDiff	0.24 (±0.04)	0.06 (±0.05)	0.14 (±0.06)	0.03 (±0.05)	0.09 (±0.05)	0.0 (±0.0)
RelativeChangeValue	0.1 (±0.18)	0.05 (±0.16)	0.05 (±0.08)	0.0 (±0.0)	0.1 (±0.15)	0.0 (±0.0)
RelativeFreqTime	0.31 (±0.17)	0.0 (±0.0)	0.07 (±0.12)	0.0 (±0.0)	0.05 (±0.16)	0.0 (±0.0)
ResultMeasuredValue	0.39 (±0.11)	0.08 (±0.08)	0.16 (±0.09)	0.02 (±0.04)	0.11 (±0.08)	0.0 (±0.0)
SdDevBL	0.31 (±0.14)	0.03 (±0.05)	0.07 (±0.08)	0.01 (±0.03)	0.07 (±0.07)	0.0 (±0.0)
SdDevChangeValue	0.24 (±0.09)	0.0 (±0.0)	0.08 (±0.07)	0.0 (±0.0)	0.09 (±0.12)	0.0 (±0.0)
SdDevResValue	0.43 (±0.12)	0.08 (±0.1)	0.15 (±0.1)	0.02 (±0.05)	0.14 (±0.11)	0.0 (±0.0)
SdErrorChangeValue	0.11 (±0.18)	0.05 (±0.16)	0.03 (±0.11)	0.0 (±0.0)	0.0 (±0.0)	0.0 (±0.0)
SubGroupDescription	-	-	-	-	-	-
TimePoint	0.36 (±0.05)	0.06 (±0.05)	0.18 (±0.1)	0.02 (±0.05)	0.14 (±0.1)	0.0 (±0.0)
Title	0.56 (±0.05)	0.17 (±0.09)	0.32 (±0.1)	0.07 (±0.1)	0.21 (±0.14)	0.0 (±0.0)
analysesHealthCondition	0.87 (±0.02)	0.14 (±0.08)	0.59 (±0.05)	0.06 (±0.1)	0.57 (±0.1)	0.0 (±0.0)
Total Micro F_1 Score	0.47 (±0.06)	0.1 (±0.05)	0.27 (±0.05)	0.04 (±0.06)	0.22 (±0.08)	0.0 (±0.0)
